# Trimethylamine-N-oxide depletes urea in a peptide solvation shell

**DOI:** 10.1073/pnas.2317825121

**Published:** 2024-03-27

**Authors:** Mazin Nasralla, Harrison Laurent, Oliver L. G. Alderman, Thomas F. Headen, Lorna Dougan

**Affiliations:** ^a^School of Physics and Astronomy, University of Leeds, Leeds LS2 9JT, United Kingdom; ^b^Disordered Materials Group, ISIS Neutron and Muon Source, Rutherford Appleton Laboratory, Didcot OX11 0QX, United Kingdom

**Keywords:** osmolyte, protein stability, denaturation, solvation, neutron diffraction

## Abstract

Trimethylamine-N-oxide (TMAO) and urea are metabolites that are used by some marine animals to maintain their cell volume in a saline environment. Urea is a well-known denaturant, and TMAO is a protective osmolyte that counteracts urea-induced protein denaturation. TMAO also has a general protein-protective effect, for example, it counters pressure-induced protein denaturation in deep-sea fish. These opposing effects on protein stability have been linked to the spatial relationship of TMAO, urea, and protein molecules. It is generally accepted that urea-induced denaturation proceeds through the accumulation of urea at the protein surface and their subsequent interaction. In contrast, it has been suggested that TMAO’s protein-stabilizing effects stem from its exclusion from the protein surface, and its ability to deplete urea from protein surfaces; however, these spatial relationships are uncertain. We used neutron diffraction, coupled with structural refinement modeling, to study the spatial associations of TMAO and urea with the tripeptide derivative glycine–proline–glycinamide in aqueous urea, aqueous TMAO, and aqueous urea–TMAO (in the mole ratio 1:2 TMAO:urea). We found that TMAO depleted urea from the peptide’s surface and that while TMAO was not excluded from the tripeptide’s surface, strong atomic interactions between the peptide and TMAO were limited to hydrogen bond donating peptide groups. We found that the repartition of urea, by TMAO, was associated with preferential TMAO–urea bonding and enhanced urea–water hydrogen bonding, thereby anchoring urea in the bulk solution and depleting urea from the peptide surface.

## Significance

Proteins are life-critical molecules whose function is typically dependent on a conformation controlled by intramolecular and intermolecular interactions. This is well illustrated in the shark which has adapted to the salinity of seawater by retaining the metabolite urea in their tissue to act as an osmolyte. This is problematic because urea, through an attractive interaction with protein, accumulates at its surface disrupting the protein’s intrapeptide bonds. The shark has evolved to produce trimethylamine-N-oxide (TMAO) to counter this urea-induced denaturation, but how this works is not well understood. Using neutron diffraction, we explore the spatial associations and interactions between peptide–water–urea–TMAO. We show that TMAO forms a hydrogen bond network with urea and water that depletes urea from the peptide surface.

The geometry of globular proteins is central to their function and to the evolution of life (1). In 1972, Anfinsen received the Nobel Prize for Chemistry for his work on ribonuclease that linked the biological conformation of proteins to the totality of their interatomic interactions and hence its amino acid sequence (2). Then, the discovery that a linear sequence of amino acids could control a protein’s native state took center stage, but Anfinsen was aware that the stability of this native form was dependent on its physiological milieu; indeed, it was by adding and removing urea from a solution of ribonuclease that Anfinsen was able to study its denaturation and renaturation. Through the 1960s and 1970s, elevated levels of urea, and a second nitrogenous metabolite, the protein-protectant, (3–5) trimethylamine-N-oxide (TMAO) were discovered in ancient classes of fish; the elasmobranchii (sharks and rays), holocephali and the coelacanths the “living fossil” (6) at a 1:2 mol ratio of TMAO:urea (4, 7). Molecular dynamics (MD) simulations on intrinsically disordered peptide sequences also suggest that TMAO eliminates protein–urea interactions at this ratio due to urea–peptide electrostatic and van der Waals interactions becoming unfavorable (8). This solute ratio is effective in both balancing the osmotic pressure of their tissue with seawater and maintaining their protein stability in the presence of the denaturant urea (9). Assays of elasmobranchii muscle and liver tissue find concentrations of ∼400 mmol and ∼200 mmol of urea and TMAO respectively (10) in stark comparison, the plasma of teleosts (bony fish) contains 2 to 4 mmol urea (11). Yancey et al. (12) showed that, in vitro, 400 mmol urea deactivated the white shark enzyme lactate dehydrogenase but that the addition of 200 mmol TMAO restored enzyme function (12). The underlying mechanisms of urea-induced denaturation are now largely understood, less so its counteraction by TMAO (8). TMAO has a wider protein-protective effect thought to be exhibited in bony fish where the TMAO concentration in their muscle tissue correlates strongly with the hydrostatic pressure at the depth of their capture (13), leading to the hypothesis that TMAO is counteracting pressure denaturation of proteins in deep-sea fish (13, 14).

**Fig. 1.**
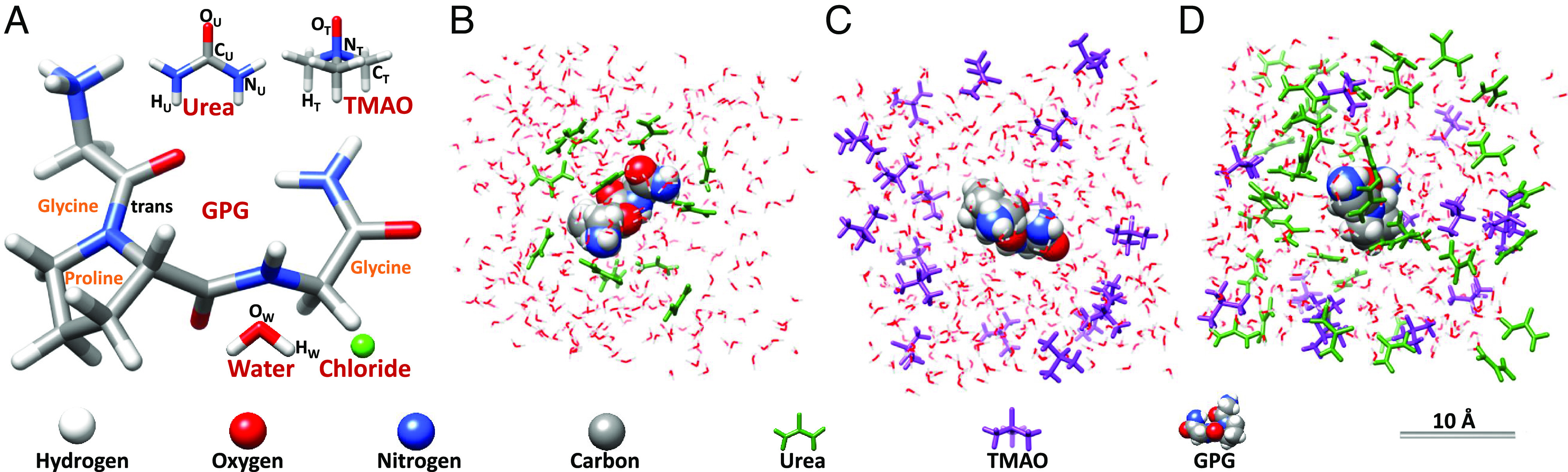
The molecular components of the solutions studied, and illustrations of some proposed solvation models from the literature. (*A*) The molecules studied in the mole ratios: 1:6:58, GPG:urea:water, 1:3:58, GPG:TMAO:water, and 1:3:6:58, GPG:TMAO:urea:water. The atoms are described by the elemental colors: carbon: gray, hydrogen: white, nitrogen: blue, oxygen: red. (*B*) illustrates the dry urea globule (15) model of a peptide in aqueous urea. (*C*) illustrates the exclusion of TMAO molecules from the surface of a peptide (16). (*D*) shows a model of urea depletion from the peptide surface (8). The spatial association of urea–peptide (*B*) in aqueous urea is well established, less so TMAO–peptide in aqueous TMAO (*C*) and urea–peptide in aqueous urea–TMAO. Here, we study the binary and tertiary solution systems shown in *B*–*D* and test the spatial models described. In *B*–*D*, GPG is represented by a 100% van der Waals model, urea and TMAO molecules are colored green and purple respectively, and water molecules are portrayed by a wire form.

The spatial association of urea, TMAO, and protein feature prominently in the mechanisms that are proposed to control protein stability (Fig. 1) (8, 15–18). Urea is thought to denature protein by accumulating at the protein–water interface (Fig. 1*B*), due to electrostatic and van der Waals interactions, where it forms hydrogen bonds with the peptide’s amide and carbonyl groups, destabilizing the peptide’s native form (15, 19–22). There is some consensus that TMAO is excluded from protein (and lipid) surfaces (16, 23–26) (Fig. 1*C*). Bolen and Baskakov (16) describe the tendency of TMAO, and other protective osmolytes, to be excluded from peptide surfaces as conducive to an osmophobic effect, a thermodynamic force that they argue is analogous to the hydrophobic effect in folding protein such that peptide exposure to a protectant-rich solution is reduced. Alternatively, it is argued that TMAO drives protein toward its native state by preferentially interacting with the folded form (18, 27, 28). Smolin and Voloshin et al. (29, 30) in MD simulations of the solvation shell of the protein staphylococcal nuclease, found that TMAO (in the TMAO:urea mole ratio 1:2) had no effect on urea–peptide association. Kokubo et al. (31), in an MD simulation of deca-alanine in aqueous TMAO–urea (mole ratio 1:2), found no evidence for the disruption of urea–peptide interactions. Similarly, Lin et al. (32), using densimetry and refractometry of RNase in aqueous urea–TMAO, found that addition of TMAO had no effect on the interaction of urea with the protein. Ganguly et al. (8), using osmometry and an MD simulation, observed urea depletion in the solvation shells of polyalanine and a protein fragment when TMAO was added to aqueous urea at the mole ratio 1:2 TMAO:urea.

Meersman et al. (33, 34) used neutron diffraction (35) to study a ternary solution of aqueous urea and TMAO, and found evidence for a urea–TMAO hydrogen-bonded complex that they suggested was responsible for TMAO’s counteraction of urea-induced protein denaturation. Steinke et al. (36) used neutron diffraction to study a solution of the amide derivative of the tripeptide Gly–Pro–Gly, glycyl-L-prolyl-glycinamide.HCl (GPG-NH_2_.HCl), hereafter GPG, in aqueous urea (in the mole ratio 1:4:58 for GPG:urea:water) and confirmed that urea molecules displayed a strong affinity for the peptide’s nitrogenous groups. GPG has been the subject of structural studies (36–41) due to its simplicity, the natural abundance of its amino acid residues, and the preponderance of Gly and Pro residues in β-turns (42), a secondary structure implicated in the nucleation of protein folding (43, 44). GPG features all of the nitrogen groups found on peptide backbones and a 4-carbon heterocyclic ring in the proline residue that provides hydrophilic and hydrophobic interaction sites for urea and the amphiphilic TMAO molecule (Fig. 1*A*).

Inspired by Steinke et al.’s study, we used neutron diffraction to study the interactions of the tripeptide GPG as a simplified model system of a globular protein surface exposed to a changing solute environment of the denaturant urea, and the protein-protectant trimethylamine-N-oxide (TMAO) in water. In particular, we focused on the spatial association of peptide molecules with urea and TMAO molecules in binary aqueous solutions of urea, TMAO, and mixed urea–TMAO in the mole ratios: i) 1:6:58 GPG:urea:water, ii) 1:3:58 GPG:TMAO:water, and iii) 1:3:6:58 GPG:urea:TMAO:water, at 298 K, 1 bar (RTP). Fig. 1 illustrates the molecular components of the solutions and some of the proposed peptide solvation structures that our experiment was designed to test (Fig. 1*B*–*D*). The tripeptide, GPG, was used here because more complex globular proteins are currently beyond the scope of empirical modeling routines used to interpret neutron diffraction studies, due to computational limitations (45). Previous results using EPSR are largely constrained to single amino acids (46, 47), dipeptides (48), tripeptides (36, 49) and 5-mer peptides (50). The largest EPSR-based study of a polypeptide sequence of which we are currently aware was performed on the 10 residue “miniprotein” CLN025 (51). Unfortunately, aqueous folded globular proteins contain within them too many partial structure factors for meaningful deconvolution with EPSR, and the increase in available conformations which would be present in unfolded proteins caused by large concentrations of denaturants like urea would certainly increase this complexity yet further. Using neutron diffraction with isotopic substitution, and computational modeling, we set out to test the hypotheses that i) TMAO depleted urea from the surface of the peptide, and ii) that TMAO was excluded from the peptide surface. The results we report here, particularly with respect to the effect of TMAO on the urea–peptide association, are significant.

## Results

The composition and structure of the peptide’s solvation shell (those molecules within ∼6 Å of a peptide atom) and the structure beyond (the bulk solution) are presented in succession through radial distribution functions [g(r)]. These functions describe the local density of the atoms and molecules of interest normalized by the bulk density as a function of their atomic separation, r.

### The Peptide Solvation Shell.

**Fig. 2.**
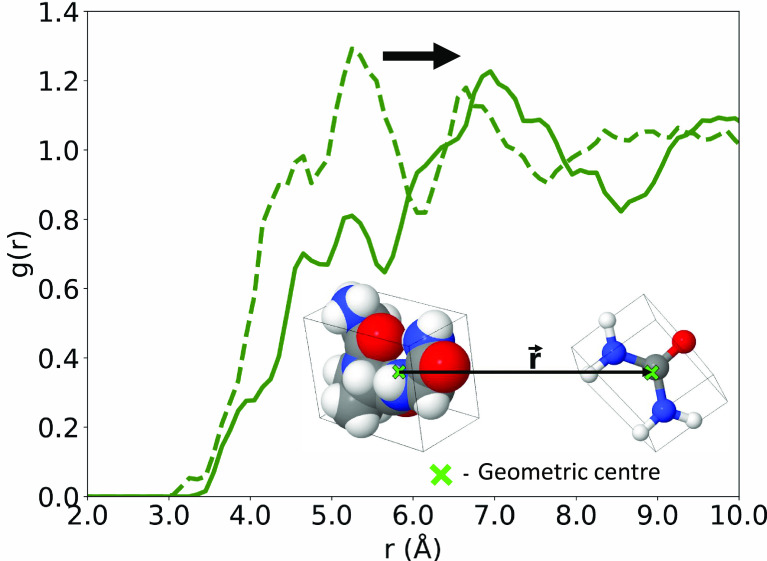
The radial distribution function (RDF) of urea molecules around the geometric center of GPG peptide molecules in samples of GPG in aqueous urea (
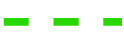
) and GPG in aqueous urea–TMAO (
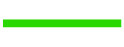
). The addition of TMAO is associated with an outward shift in the urea RDF. *Inset*: The geometric centers of GPG and urea molecules represented within 3-D boxes. r, is defined in reference to the centers of molecular geometry.

Fig. 2 describes the radial distribution function (RDF) of urea molecules around the centroid, or center of geometry (COG) of GPG, in aqueous urea (dashed green line) and in aqueous urea–TMAO (solid green line). The RDFs were derived from molecular simulations (*SI Appendix*, Figs. S1–S3) refined to be consistent with the diffraction measurements. The x, y, and z coordinates of molecules in the simulations were averaged in each configuration of a molecular trajectory to derive a RDF by the center of molecular geometry.

Fig. 2 shows that in aqueous urea–TMAO, there is an outward shift in the RDF of urea (below 6 Å from the COG of GPG) that is indicative of the depletion of urea molecules from the peptide surface.

Analysis of the x,y,z coordinates of the molecular species showed that in a sphere of radius of 6 Å from the COG of the GPG molecule, the number of urea molecules was reduced by 42% on the addition of TMAO, while the number of water molecules increased by 6%. There was no significant change in the spatial association of GPG–TMAO as urea and TMAO concentrations were changed (*SI Appendix*, Figs. S4 and S5).

#### The urea preference ratio.

We have shown that the distribution of urea in the peptide solvation shell is dependent on the presence of TMAO (Fig. 2) but it also shows a strong atomistic variation in the affinity of each peptide atom for urea, water, and TMAO atoms. To quantify this atomistic variation in the molecular environs of each peptide atom, we calculate a urea preference ratio (UPR) (36, 52), that is the ratio of the coordination (Eq. **5**) of GPG atom X around an atom Y of urea, (n$\begin{array}{c} X_{\mathrm{GPG}}\\ Y_{\mathrm{urea}} \end{array}$) to the coordination of that GPG atom X around an atom Z of water (Eq. **1**). We then compare the UPR, atom by atom, in the aqueous urea and aqueous urea–TMAO samples. This inverse coordination number ratio allows for a meaningful comparison of GPG atomic affinity for urea and water species that have different concentrations in solution. The coordination number ratio in Eq. **1** is calculated at the average of the first gX_GPG_-Y_urea_(r) and gX_GPG_-Z_water_(r) minima (atomic bond lengths) so that each GPG atom’s UPR index is evaluated at a single appropriate distance. We deduct 1 from the ratio in Eq. **1** so that a positive UPR describes a GPG atom with a greater affinity for urea than water, and vice versa. We calculate a TMAO preference ratio (TPR) by substituting Y_TMAO_ for Y_urea_ in Eq. **1**

[1] $$\begin{array}{c} UPR=\frac{n\begin{array}{c} X_{\mathrm{GPG}}\\ Y_{\mathrm{urea}} \end{array}}{n\begin{array}{c} X_{\mathrm{GPG}}\\ Z_{\mathrm{water}} \end{array}}-1. \end{array}$$

**Fig. 3.**
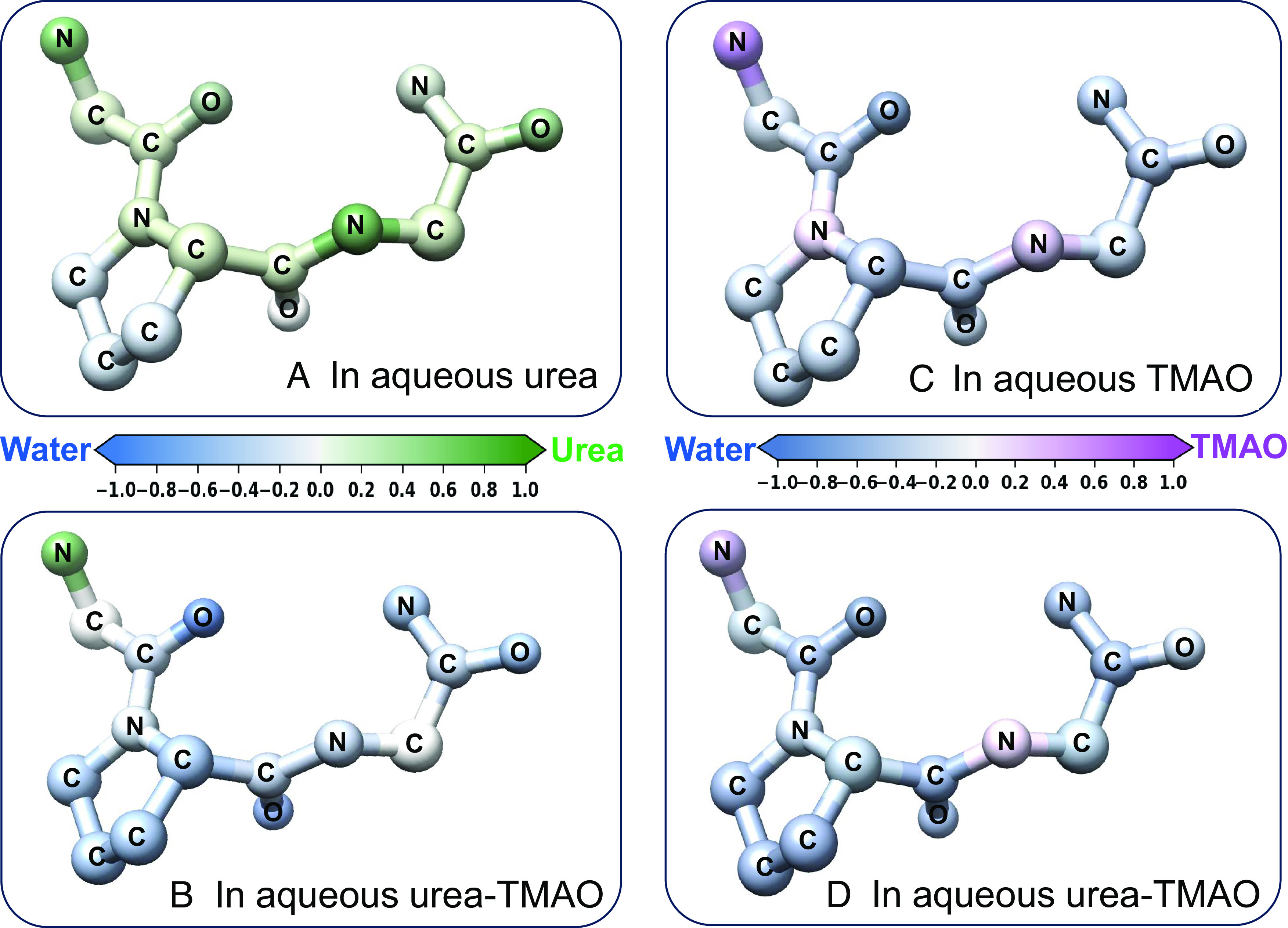
The coordination of atoms in the peptide backbone by urea, TMAO and water molecules in aqueous samples of GPG-urea, GPG-TMAO and GPG–urea–TMAO. The data were derived from simulations refined against the neutron diffraction data. A urea preference ratio, measuring atomic coordination of each peptide atom to urea, relative to water, is mapped to the GPG molecule through the water–urea (blue-green) color bar in the aqueous urea (*A*) and aqueous urea–TMAO (*B*) samples. Similarly, a TMAO preference ratio is mapped to peptide atoms through the water–TMAO (blue-purple) color bar in the aqueous TMAO (*C*), and aqueous urea–TMAO (*D*) samples.

In Fig. 3 *A* and *B*, we show in atomistic detail, GPG’s relative preference for urea and water in aqueous solutions of urea a) and aqueous urea–TMAO b) by color-mapping each atom’s UPR index through a green-blue color bar. Similarly, Fig. 3 *C* and *D* describe GPG’s atomic preference for TMAO or water through a purple-blue color map. The illustration of the GPG molecule presented in Fig. 3*A* indicates that in aqueous urea, urea dominates the solvation of the peptide, particularly around the amide group, the NH_3_ end-group and the carbonyl groups. In contrast, in aqueous urea–TMAO (Fig. 3*B*), with the exception of the NH_3_ end-group, the peptide atoms are solvated predominantly by water molecules. The addition of TMAO leads to a breakdown in peptide atom–urea coordination. Even at the polar NH_3_ group, urea dominance is reduced.

Fig. 3 *C* and *D* show that water dominates the solvation of the peptide’s atoms in aqueous TMAO except for the regions around the nitrogen groups. Fig. 3 *C* and *D* appear broadly similar suggesting little variation in the spatial association of TMAO–peptide/water–peptide in the aqueous TMAO and aqueous urea–TMAO solutions. *SI Appendix*, Table S1 documents the numerical UPR and TPR indices and *SI Appendix*, Figs. S7–S9 present additional atomic interactions between the peptide and its solvation shell in the different solution systems. Subsequent error analysis involving experimental repeats reproduced these results (*SI Appendix*, Fig. S10 and Table S2) and variable urea coordination of the carbonyl atoms that we suggest is linked to the structural conformation and geometry of the GPG, urea, and water molecules.

### The Bulk Solution.

**Fig. 4.**
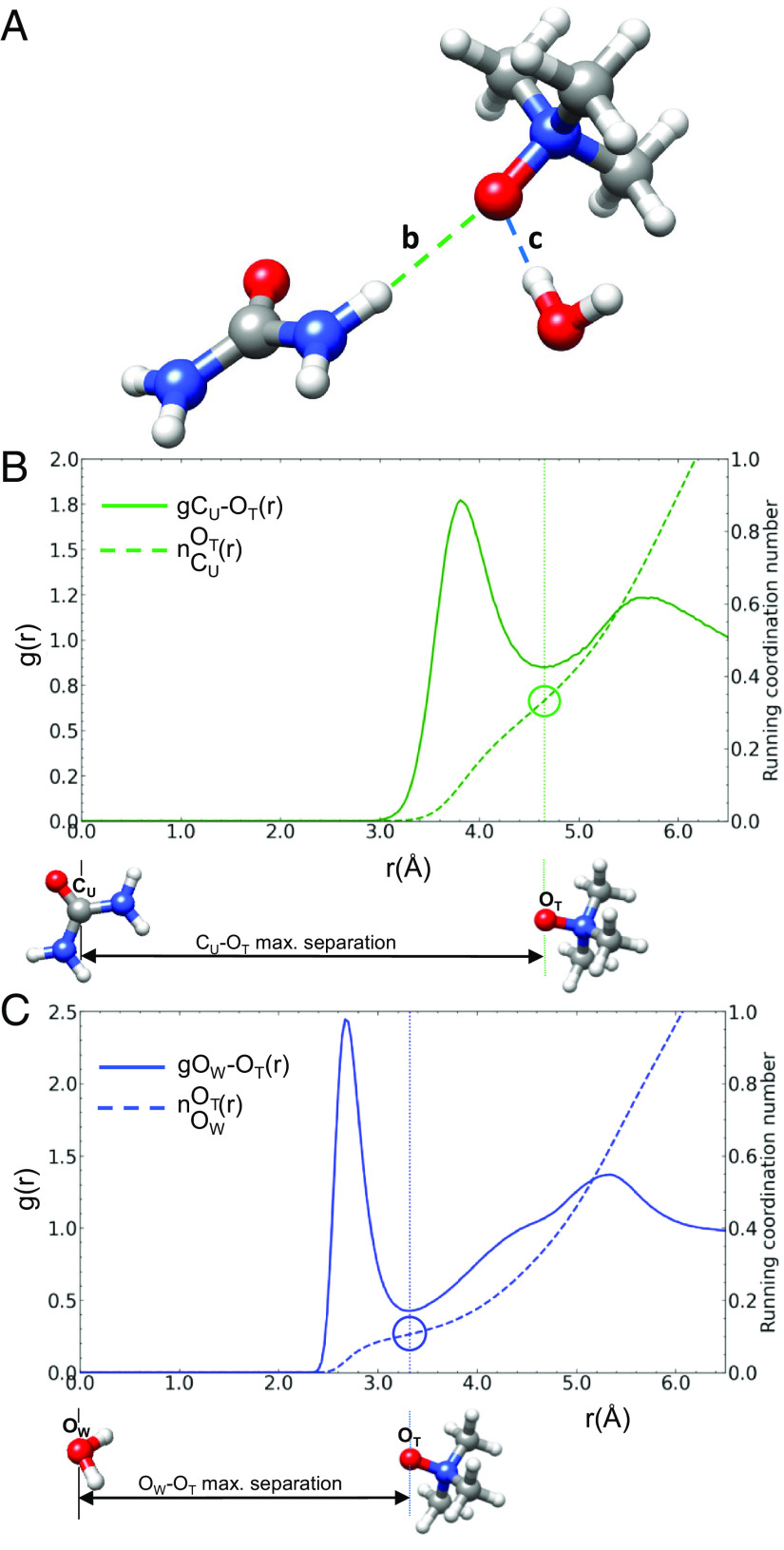
A comparison of urea–TMAO and water–TMAO interactions shows that TMAO molecules are three times as likely to complex urea molecules compared to water molecules. (*A*) describes the comparison by illustrating a TMAO molecule accepting hydrogen bonds from urea (*B*) and water molecules (*C*). In (*B*), the solid green line (
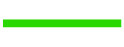
) describes the gC_U_-O_T_(r) and the dashed green line (
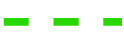
) describes the average coordination of $C_{U}$ by O_T_ as their separation varies. In (*C*), the solid blue line (
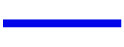
) describes the gO_W_-O_T_(r) and the dashed blue line (
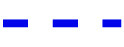
) describes the coordination of O_W_ atoms by O_T_ atoms. The dotted green (
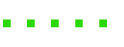
) and blue (
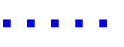
) vertical lines mark the maximum separation of C_U_ and O_T_ atoms, and O_W_ and O_T_ atoms in the urea–TMAO and water–TMAO complexes. The green and blue circles mark the coordination of C_U_ by O_T_, and the coordination of O_W_ by O_T_ at the maximum separation of the complexes.

To provide more detail on the extent of TMAO’s interactions with urea and water, Fig. 4 compares some of their pertinent RDFs (g(r)). Fig. 4*B* describes the urea–TMAO hydrogen bond through a RDF between the central carbon atom of urea (C_U_) and the oxygen atom of TMAO ($O_{T}$), gC_U_-O_T_(r) (solid green line). The gC_U_-O_T_(r) peaks at the mean separation of $C_{U}$-$O_{T}$ atoms (3.81 Å), and the first minima (4.65Å) marks the maximum separation of $C_{U}$-$O_{T}$ atoms in urea–TMAO complexes. The dashed green line in Fig. 4*B* describes the coordination number $n\begin{array}{c} O_{T}\\ C_{U} \end{array}$(r), the average number of O_T_ atoms around a central $C_{U}$ atom as a function of their separation, r. The average coordination of $C_{U}$ atoms by O_T_ is 0.33. Fig. 4*C* describes the water–TMAO hydrogen bond through the pair correlation of the oxygen atom of water ($O_{W}$) and the oxygen atom of TMAO ($O_{T}$), gO_W_-O_T_(r) (solid blue line). The dashed blue line in Fig. 4*C* describes the coordination number $n\begin{array}{c} O_{T}\\ O_{W} \end{array}$(r), the average number of O_T_ atoms around the central $O_{W}$ atom as a function of r. The average coordination of $O_{W}$ atoms by O_T_ was 0.11. The ratio, $\frac{n\begin{array}{c} O_{T}\\ C_{U} \end{array}}{n\begin{array}{c} O_{T}\\ O_{W} \end{array}}$, measures the relative propensity for TMAO to hydrogen bond urea molecules compared to water molecules. In 1:3:6:58 GPG:urea:TMAO:water, TMAO is 3.0 times as likely to hydrogen bond urea molecules as water. This is a significant result as the TMAO–water hydrogen bond is itself relatively strong (14) (*SI Appendix*, Fig. S11*A*).

**Fig. 5.**
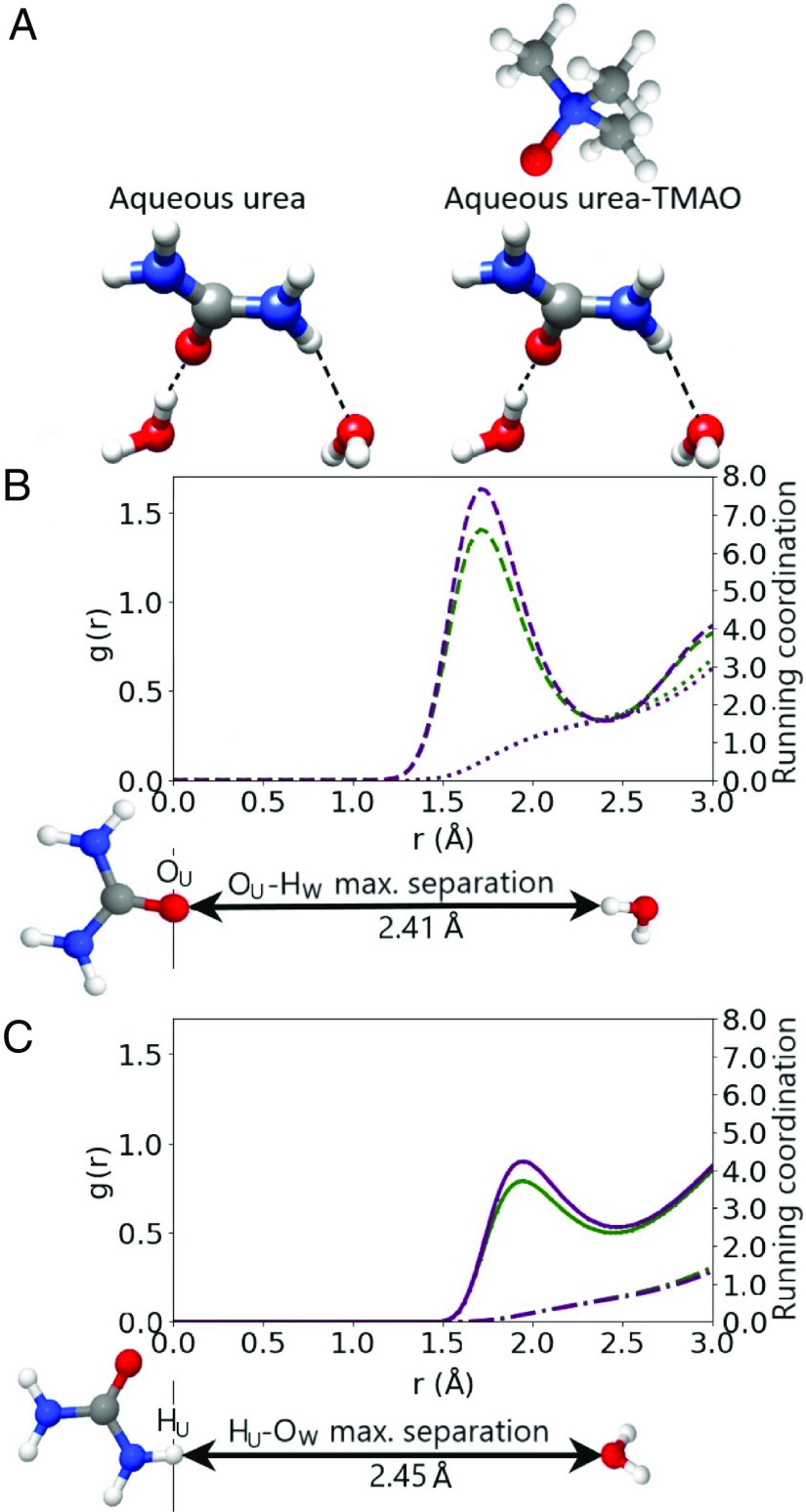
A comparison (*A*) of the urea–water network in aqueous urea and aqueous urea–TMAO. (*B* and *C*) show that the coordination of urea by water is unchanged by the addition of TMAO. (*B*) describes the gO_U_-H_W_(r) in aqueous urea–TMAO (
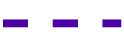
), and aqueous urea (
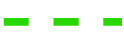
) respectively. The coordination of the central O_U_ atom by H_w_ is described by dotted lines in aqueous urea–TMAO (
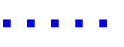
) and aqueous urea (
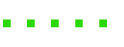
) respectively. (*C*) describes the gH_U_-O_W_(r) in aqueous urea–TMAO (
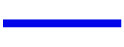
) and aqueous urea (
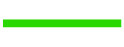
) respectively. The coordination of the H_U_ atom by O_W_ is described by a dash-dotted line in aqueous urea–TMAO (
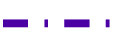
) and aqueous urea (
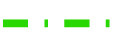
) respectively. In *B* and *C* the lines marking the coordination almost overlie each other, showing that the coordination is unchanged by the addition of TMAO.

To demonstrate the effect of TMAO on the urea–water hydrogen bond network, Fig. 5 describes that network in GPG–urea–water and GPG–urea–TMAO–water through the RDFs of the urea–water hydrogen bonds and their coordination numbers. Fig. 5*A* illustrates the two types of hydrogen bond in a urea–water network: i) the O_U_-H_W_, and ii), the H_U_-O_W_ interactions. Fig. 5*B* describes the gO_U_-H_W_(r) in aqueous urea (dashed green line), and in aqueous urea–TMAO (dashed purple line). Fig. 5*C* describes the gH_U_-O_W_(r) in aqueous urea (solid green line), and in aqueous urea–TMAO (solid purple line). The peaks in the gO_U_-H_W_(r) (1.71Å, Fig. 5*B*) are larger and more defined, than the peaks in the gH_U_−O_W_(r) (1.94 Å, Fig. 5*C*), and the O_U_-H_W_ bond is shorter than the H_U_-O_W_ bond. This indicates that the O_U_-H_W_ is the stronger urea–water interaction. The gO_U_-H_W_(r), gH_U_-O_W_(r) are described by more defined first peaks in the presence of TMAO that suggests a higher proportion of hydrogen bonding between urea and water. The coordination of urea atoms by water atoms ($n\begin{array}{c} H_{W}\\ O_{U} \end{array}$(r), $n\begin{array}{c} O_{W}\\ H_{U} \end{array}$(r)) is unchanged by the addition of TMAO, evidenced by the overlying n(r) (the green and purple dotted lines in Fig. 5 *B* and *C*) yet water’s mole fraction of solution is reduced in GPG–urea–TMAO–water ($\frac{58}{68}$, compared to $\frac{58}{65}$ in GPG–urea–water). This is notable because in the GPG–urea–TMAO–water sample, water is competing with TMAO to hydrogen bond with urea. TMAO preferentially binds urea and enhances hydrogen bonding between urea–TMAO–water molecules, and in so doing replaces the peptide from the urea molecules coordination shell.

## Discussion

It is well established that urea denaturation proceeds after urea accumulates at the protein surface due to differential enthalpic interactions between urea–protein and urea–water molecules (15, 19–22). We also report strong peptide–urea interactions. Regarding the TMAO-induced depletion of urea at the peptide surface, it was our observation that this was driven by the complexing of urea with a TMAO–water hydrogen bond network that would have a universal effect on urea–peptide association, whatever the peptide’s size and complexity. The complexing of urea with a TMAO–water hydrogen bond network has been observed in inelastic x-ray scattering measurements, where strong TMAO–water interactions result in an indirect TMAO–urea association (53). While we can then translate the urea–peptide, and TMAO-induced urea depletion to proteins, we do not translate the TMAO–GPG spatial association to proteins, as well as other structured biomolecules where TMAO is observed to have a stabilizing effect such as riboswitches and DNA hairpins (54, 55). We had expected that GPG would be completely solute accessible, but we found that its structural interactions were influenced strongly by steric effects such that the gO_i_-H_U_(r) showed significant variation between carbonyl groups (*SI Appendix*, Fig. S8 and Table S2). This is understandable in terms of the spatial restrictions imposed on urea and carbonyl atoms by the peptide’s conformation. With respect to TMAO’s interactions with GPG, we found that TMAO interacted strongly with hydrogen bond donating end-groups and very weakly with the carbonyl groups (*SI Appendix*, Figs. S7 and S8). There are two hypotheses for TMAO–protein interactions. One holds that TMAO is excluded from the protein surface (16), and the other that TMAO interacts preferentially with polar atoms on the protein surface stabilizing the native form (18, 27, 28). It is possible to find support for both models in our results. Given the extent of steric hindrance toward solute molecules in the tripeptide GPG, caution should therefore be taken in translating the spatial association of TMAO with the tripeptide to large proteins. Steric hindrance of a trimethylated TMAO molecule together with its ready acceptance of hydrogen bonds from water may lead to a different structural outcome for large proteins.

Our experimental objective was to test the solvation models (Fig. 1) proposed for protein in aqueous TMAO and aqueous urea–TMAO, and to derive the mechanisms by which TMAO might affect any structural change. The results shown in Fig. 2 and Fig. 3 *A* and *B* demonstrate a TMAO-driven depletion of urea from the peptide surface. Our results show that TMAO was not excluded from the tripeptide surface (Fig. 2 and *SI Appendix*, Figs. S7–S9) but that the large peaks in the peptide–TMAO g(r) were restricted to the hydrogen bond donating end NH_3_ and NH_2_ caps. Water exhibited no such preference. *SI Appendix*, Fig. S7 shows that urea also interacted very strongly with these end groups, but that unlike TMAO, urea also interacted strongly with the peptide’s carbonyl groups (*SI Appendix*, Fig. S8). TMAO also interacted weakly with proline’s heterocyclic ring through a hydrophobic association. This result is expected, as urea contains both hydrogen bond donating and accepting groups, whereas TMAO only contains one strong hydrogen bond acceptor (33); hence, it is unlikely to interact strongly with hydrogen bond accepting carbonyl groups. These fundamental enthalpic interactions inform how we might view TMAO’s interaction with a larger globular protein, but the result that TMAO was not excluded from the tripeptide’s surface should not necessarily be translated to larger protein surfaces. Statistical thermodynamics results suggest that stabilizing osmolytes such as TMAO are more excluded from the native state than the denatured state, hence promoting native stability (56). As our GPG model is sufficiently small such that it is entirely solvent exposed, and hence effectively denatured, it is likely that an osmolyte such as TMAO would have a more observable exclusion from the surface were a larger folded model to be used. *SI Appendix*, Fig. S12 showed that in aqueous solutions of urea, TMAO, and urea–TMAO, GPG exists in largely open loops, and linear chains. In comparison, globular proteins are characterized by a compact hydrophobic core, around which peptide strands interact in a variety of folded structures (57). In contrast, if TMAO is effective in depleting urea from a small open peptide structure, we can translate this to a globular protein, particularly if the effect is, as we argue here driven by interactions between TMAO–urea–water molecules in the bulk solution.

**Fig. 6.**
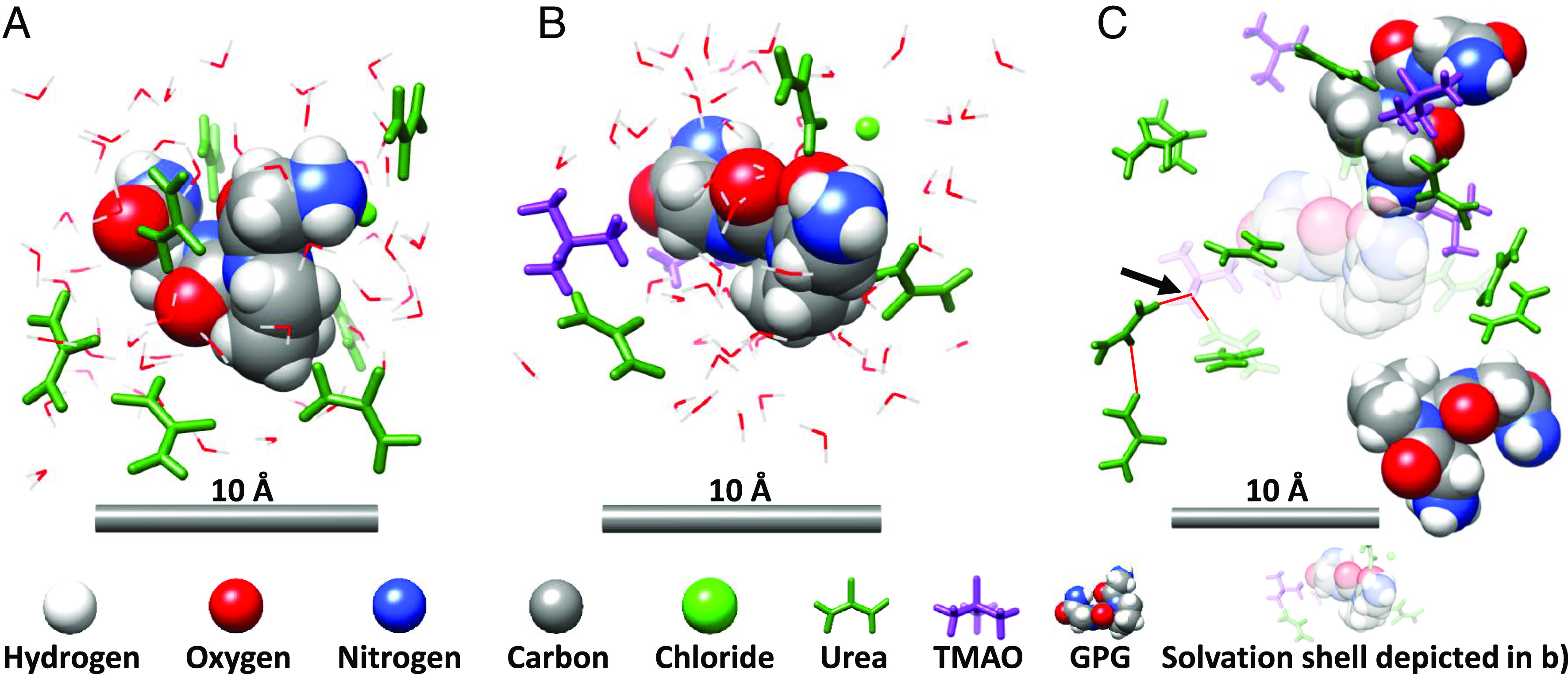
Representative solvation structures drawn from the structural refinement modeling of the diffraction data that pertain to the peptide in aqueous urea (*A*) and aqueous urea–TMAO (*B* and *C*). (*A*) shows the urea-rich surface of GPG tripeptide in aqueous urea. (*B*) shows the surface of a GPG tripeptide that is now relatively depleted in urea, in a solution of aqueous urea–TMAO, and (*C*) shows the bulk-solution further surrounding the GPG interface of the structure in (*B*) in aqueous urea–TMAO. The arrow marks a TMAO molecule entrapping urea in a hydrogen bond network. Urea and TMAO molecules are colored green and purple throughout, while water molecules are described by a wire form which has been hidden in (*C*) to aid visualization.

To give a clear picture of the solvation structures, and the mechanism underlying TMAO’s counteraction of urea, Fig. 6 *A* and *B* provides representative examples of peptide molecules, from simulations that have been refined by the diffraction measurements, in aqueous urea, and aqueous urea–TMAO. These examples are consistent with the statistical analysis presented in Figs. 2 and 3 and *SI Appendix*, Figs. S7–S9. Fig. 6 *A* and *B* pictures molecules within a ∼6 Å radius of the GPG molecule’s geometric center, effectively capturing the GPG molecule and its coordination shell. In Fig. 6*C*, the GPG molecule and solvation shell described in Fig. 6*B* is made transparent as we zoom out to focus on molecules up to ∼10 Å from the COG of GPG so as to highlight the role of a TMAO molecule in anchoring urea into a hydrogen bond network in the bulk solution.

The solvation structures in Fig. 6 show the spatial associations of peptide–urea–TMAO molecules. We now set out to explain how TMAO’s molecular interactions may underlie these spatial associations and TMAO’s protein-protective effects.

Atomistically detailed TMAO preference ratios (Fig. 3 *C* and *D*) and detailed g(r) (*SI Appendix*, Figs. S7 parts *C*, *I*, and *L*, S8, and S9) show that TMAO’s strong interactions with the tripeptide were limited to the peptide’s hydrogen bond donating nitrogen groups, particularly its amide, and terminal NH_3_ groups. This result is in disagreement with the findings of an MD study which found that the amide–TMAO interaction was highly unfavorable (58). The gO_i_-H_W_(r) (*SI Appendix*, Fig. S8 *C*, *F*, and *I*) feature strong peaks in the g(r) at ∼ 1.7 Å indicative of strong carbonyl–water interactions, whereas the comparative gO_i_-H_T_(r) feature shoulders rather than peaks, indicating a much weaker carbonyl–TMAO interaction. The GPG tripeptide, has 1 hydrophobic entity, the heterocyclic, proline ring. The gH_T_-C_r_(r) shown in *SI Appendix*, Fig. S13 shows a weak but noticeable increase in the density of TMAO hydrogen atoms at ∼3 Å from the carbon atoms (Cr) of the proline ring. This peptide–TMAO dispersion interaction was only detectable around the proline residue. In contrast to the limited number of strong peptide–TMAO interactions, the gO_T_-H_W_(r) and gO_T_-H_U_(r) (*SI Appendix*, Fig. S11) show that TMAO strongly accepts hydrogen bonds from water and urea molecules. In the case of a globular protein with proportionately fewer hydrogen bond donors and with the effects of steric hindrance toward an amphiphilic molecule, the differential enthalpic interaction between TMAO–protein, and TMAO–water, may lay the basis for TMAO’S exclusion from larger protein surfaces.

There is considerable evidence that hydrophobic interactions lead to the burial of nonpolar amino acids in the protein-folded core and the simultaneous decoration of a solvent-exposed surface with hydrophilic residues and groups (44). This observation is compatible with the hypothesis that TMAO drives protein toward its native state by exhibiting enhanced enthalpic interactions with the folded form (27, 28). Our results do not support this hypothesis. We demonstrated that while TMAO strongly accepts hydrogen bonds from the tripeptide, it formed very weak interactions with the tripeptide’s hydrophilic carbonyl groups. We also showed that TMAO exhibits a weak attractive interaction with the tripeptide’s hydrophobic proline ring. This agrees with a previous molecular dynamics study of neopentane as a model hydrophobic entity in an aqueous urea and TMAO solution, where it was observed that TMAO preferentially associated with neopentane by orienting its methyl groups toward the surface such that its NO group could form hydrogen bonds with the surrounding water (59). Taken together, these findings do not support the hypothesis that TMAO promotes protein-folding by enthalpic interactions with what would be a limited number of atom types. The lack of enthalpic stabilization by TMAO is also observed in differential scanning calorimetry results on RNase A as a model protein. Here, it is demonstrated that stabilizing agents including TMAO do not affect the magnitude of the enthalpy change of unfolding at the melting temperature, and hence, their stabilization is likely to be entropic (60).

Our results suggest a mechanism by which TMAO depletes urea at the peptide surface to counteract urea-induced protein denaturation. MD simulations suggest that urea molecules partition toward the protein surface due to favorable direct interactions with the protein molecule (15, 21). We find that TMAO acts both directly and indirectly on urea through preferentially hydrogen bonding urea (Fig. 4) and by cooperatively enhancing the hydrogen bond structure (61, 62) of water–urea that prevents the accumulation of urea at the peptide surface. Fig. 6 draws on representative model peptide solvation structures from our studies to illustrate both the depletion of urea at the surface of a GPG tripeptide and the formation of a hydrogen bond network in the bulk solution that anchors urea in the bulk solution depleting urea from the peptide’s surface.

The results we report here will serve as a benchmark for MD studies where there is much discussion about the sensitivity of MD results to the forcefields adopted in urea–TMAO MD simulations (17). We note that the empirical potential is refined purely against structural data, and its reliability for predicting thermodynamic and/or kinetic data from simulations has not been fully tested.

We showed through an analysis of the coordination of urea and water by TMAO (Fig. 4), that in the aqueous urea–TMAO sample, TMAO was 3.0 times as likely to hydrogen bond urea molecules than water. Analysis of the urea–water hydrogen bond network (Fig. 4) showed that this network remained entirely intact in the presence of TMAO. Taken together, we can conclude that TMAO enhances the overall hydrogen bond network in solution.

It was Anfinsen’s hypothesis that a protein’s biological conformation was controlled by thermodynamic interactions between its amino acid residues and that the biological conformation was stable in a prescribed physiological milieu. The nature of the interactions between key metabolites in the cellular environment of some marine animals, and their interactions with the peptide building blocks of proteins has been the subject of this study. We have illustrated the interactions between urea and peptide atoms that Anfinsen put to such good effect, as other researchers have done (15, 19, 36), but we have also shown how interactions between TMAO, urea, and water, a component of the cytosol of some marine animals, might be responsible for the counteraction of urea-induced protein denaturation, and that this is associated with the depletion of urea at the peptide surface. We showed that TMAO was not excluded from the model tripeptide’s surface and while we do not make any inferences regarding TMAO’s spatial association with larger biological proteins, we have taken a step forward by revealing the nature of TMAO’s atomic interactions with the tripeptide molecule.

## Materials and Methods

### Sample Preparation.

Glycyl-L-prolyl-glycinamide.HCl (GPG.HCl) was purchased from Bachem, Switzerland. TMAO was purchased from Sigma Aldrich and urea from Thermo Fisher Scientific. Deuteriated urea (d4) and deuteriated TMAO (d9) were purchased from CK Isotopes Ltd. The six exchangeable hydrogen atoms of GPG.HCl were deuteriated by the ISIS deuteration facility. All samples were ≥98% pure with the exception of TMAO which was ≥95% pure and all were used without further purification. Immediately prior to the diffraction experiment, ∼1.5 ml aliquots of protiated and deuteriated samples were prepared by dissolving appropriate masses of crystalline sample in pure, and deuteriated water (*SI Appendix*, Table S3). The samples were transferred by syringe to flat, null scattering alloy (Ti_0.68_Zr_0.32_) cans of 1 mm thickness. The cans were then sealed with Teflon and mounted onto a sample changer.

### The Neutron Diffraction Experiment.

The sample changer was loaded into the Near and InterMediate Range Order Diffractometer (NIMROD) (63), at the ISIS neutron and muon source, the sample chamber evacuated, and the beam-line shutter opened with the sample changer rotated remotely in the beam. Each sample was in the neutron beam for up to 4 h at RTP over a period of 4 d, with neutron scattering detected through banks of ZnS-based scintillation detectors. In order to put the results on an absolute scale, a plate of null-scattering VNb alloy, 3 mm thick, with known scattering characteristics, was placed in the beam for 2 h, under identical experimental conditions. Empty TiZr null scattering alloy cans were placed in the beam for up to 4 h, and neutron scattering with an empty instrument was also measured. The data reduction package, Gudrun (64), was used to subtract the scattering from empty cans and the sample background, and to convert the “raw” counts to useful differential cross-section data. The aqueous samples, rich in the light element hydrogen, generate large inelastic scattering effects that were removed by an iterative method in Gudrun using a Top Hat width of 15 A^−1^ in the convolution method (64).

This experiment used a technique described as neutron diffraction with isotopic substitution (NDIS) (65). NDIS exploits the contrast in the scattering power of different isotopologues that generates a unique differential scattering cross-section from isotopically different but structurally, and chemically equivalent samples. The coherent scattering length of hydrogen is −3.74 fm (35), and its isotope, deuterium, exhibits a large contrast in its scattering length (6.67 fm). The resultant scattering pattern, or total Structure Factor (F(Q)) can be thought of as the weighted sum of individual partial structure factors (S(Q)) of each scattering center atom-atom correlation (α,β) (Eq. **2**). We selected mixtures of fully protiated, and deuteriated samples, and mixtures thereof, that contained up to 7 isotopic substitutions (*SI Appendix*, Table S3) noting that urea’s hydrogen atoms, and the hydrogen atoms associated with GPG’s nitrogen groups are all exchangeable. In making these isotopic substitutions, we obtain composite structure factors, weighted by the nuclides’ scattering lengths, and concentration, of the form F(S_HH_(Q) + S_HX_(Q) + S_XX_(Q)), where S_HH_ gives the partial structure factor of hydrogen nuclides, S_HX_ gives the partial structure factors of hydrogen and other nuclides, and S_XX_ gives the partial structure factor of non-hydrogen nuclides. Our experimental objective was to measure the spatial association of peptide, urea, and TMAO molecules, that is, to measure S_XX_, and this was reflected in the isotopic substitution strategy we adopted. The contribution of the S_HH_ partial structure factors was reduced by creating HD samples, where the negative and positive scattering lengths partially offset each other, thereby increasing the weighted component of S_XX_ in the scattering pattern.

[2] $$\begin{array}{c} F\left(Q\right)=\sum_{\alpha ,\beta }\left(2-\delta_{\alpha ,\beta }\right)c_{\alpha }c_{\beta }b_{\alpha }b_{\beta }\left(S_{\alpha \beta }\left(Q\right)-1\right), \end{array}$$

where c is the fractional atomic concentration, b is the nuclide’s scattering length, δ the kronecker delta, and Q the scattering vector (Eq. **3**) with dimensions of L^−1^ (reciprocal space).

[3] $$\begin{array}{c} Q=\frac{4\pi \sin(\theta )}{\lambda }, \end{array}$$

where 2θ is the angle between the incident and scattering neutrons, and λ their wavelength.

The partial structure factor (S_*αβ*_(Q)) is related to the radial distribution function (the local density of atom β around atom α as a function of r, normalized by the bulk density of β) of the atom pair: α,β by a Fourier transform (Eq. **4**),

[4] $$\begin{array}{c} S_{\alpha \beta }\left(Q\right)=\rho \int_{0}^{\infty }g_{\alpha \beta }\left(r\right)e^{\mathrm{iQr}}dr=4\pi \rho \int_{0}^{\infty }g_{\alpha \beta }\left(r\right)\frac{\sin\mathrm{Qr}}{\mathrm{Qr}}dr, \end{array}$$

where ρ is the experimentally determined atomic number density of the sample and r the atomic separation of α-β. Integration of the g(r) over the integration limits, r_*min*_ and r_*max*_, gives the coordination number (the average number of β atoms around a central α in the radial distance $r_{\min}$ up to r_*max*_ from atom α Eq. **5**)

[5] $$\begin{array}{c} n\begin{array}{c} \beta \\ \alpha \end{array}\left(r\right)=4\pi \rho c_{\beta }\int_{r_{\min}}^{r_{\max}}r^{2}g_{\alpha \beta }\left(r\right)\;dr. \end{array}$$

In a molecule containing i different atomic species, the number (N) of different atomic correlations (α-β) is given by Eq. **6**

[6] $$\begin{array}{c} N=\frac{i(i+1)}{2}. \end{array}$$

For pure water, i = 2, and N = 3(O_W_-O_W_,H_W_-H_W_,O_W_-H_W_). In the system GPG–urea–TMAO–water, we have four molecules in which we defined 26 atom types of interest. With i = 26, we would need 351 distinct isotopologues to derive the g_*αβ*_(r). The problem of inverting the diffraction data to real-space data grows significantly with sample complexity and Empirical Potential Structure Refinement (EPSR), available from the ISIS Neutron and Muon source, is a widely used structural refinement modeling system that provides a solution to this problem (66, 67). We used a priori knowledge of the components to simulate molecular models of the solution systems with user-defined molecular geometries (*SI Appendix*, Figs. S12–S15), Lennard-Jones potentials (*SI Appendix*, Tables S4–S7), and a cubic box-dimension to simulate the experimentally determined density (*SI Appendix*, Figs. S1–S3). In the case of GPG, the molecule was set up in EPSR such that the peptide bonds were fixed to be planar; otherwise, free bond rotation was allowed (*SI Appendix*, Fig. S17), and then, we refined this a priori informed simulation against the diffraction measurements obtained from experiment. NMR HSQC spectroscopy showed that ∼12% of GPG exists in cis form (proportion is invariant in water, urea, and TMAO. *SI Appendix*, Fig. S21) in agreement with previous findings (36), so in each ESPR model of the solution system 9 of the GPG molecules were set up in cis form, and 65 in trans form (*SI Appendix*, Fig. S17). After randomizing the molecules, and equilibrating the box so that it adopts the most stable configuration, the Monte Carlo (68) simulation proceeds steadily exploring intermolecular and intramolecular configurations (*SI Appendix*, Figs. S1–S3) by iteratively testing randomized atomic movements (whole molecular translations, rotations, and individual atomic movements) against the metropolis condition such that the probability of the move being accepted is ${\text{e}}^{\frac{-\Delta U}{\mathrm{kT}}}$ where kT is the Boltzmann Thermal factor, and U a combination of a Lennard-Jones potential, coulomb potential and an empirical potential (EP). After initial equilibration, the EP is derived from the difference between the measured and simulated F(Q) (based on atomic positions in the box). Over time the residual difference between the measured and simulated F(Q) is reduced (*SI Appendix*, Figs. S22–S30). A nondefault ref_intra setting (0 1) was set in EPSR to apply the full reference potential to intramolecular pairs, effectively preventing aphysical intramolecular atomic separation. In this way, an empirical potential, derived from the diffraction measurements, refines the structure in the simulation box so that it is consistent with the measured diffraction data and structural data can be extracted.

## Supplementary Material

Appendix 01 (PDF)

## Data Availability

The EPSR working directories have been deposited at the University of Leeds (https://doi.org/10.5518/1368) (69).
