# Supplementary material for: Trimethylamine-N-oxide depletes urea in a peptide solvation shell
Source: Proc Natl Acad Sci U S A. 2024 Mar 27;121(14):e2317825121. doi: 10.1073/pnas.2317825121 (PMC10998561; doi:10.1073/pnas.2317825121)
Supplement: Supplementary file 1 — Appendix 01 (PDF) [file pnas.2317825121.sapp.pdf]

Supporting Information for

Trimethylamine-N-oxide depletes urea in a peptide solvation shell

Mazin Nasralla, Harrison Laurent, Oliver L.G. Alderman, Thomas F. Headen, and Lorna Dougan

Corresponding Author: Lorna Dougan.
E-mail: l.dougan@leeds.ac.uk

This PDF file includes:

Supporting text
Figs. S1 to S30
Tables S1 to S7
SI References

Supporting Information Text

Additional experimental data will be available through the University of Leeds (DOI: https://doi.org/10.5518/1368)

Contents:

Fig. S1-S3: The EPSR simulation boxes and their molecular components for GPG-urea-water, GPG-TMAO-water and GPG-urea-TMAO-water.
Fig. S4-S5: The molecular radial distribution functions of GPG-urea, GPG-TMAO and GPG-water.
Fig. S6: The molecules, atom-types and their acronyms.
Fig. S7-S9: Peptide-solute and peptide-water intermolecular g(r) compared.
Fig. S10: A comparison of the Urea Preference Indices derived from a series of Monte Carlo simulations in EPSR that utilise a) reference Lennard Jones potential and b) an empirical potential drawn from the scattering data. Fig. S11: The $g_{O_T\text{-}H_W}(r)$, and $g_{O_W\text{-}H_W}(r)$ in aqueous urea-TMAO.
Fig. S12: The intramolecular $g_{O1\text{-}Hn2}(r)$.
Fig. S13: The $g_{HT\text{-}C_i}(r)$
Fig. S14-S17: The EPSR set-up.
Fig. S18-S20: GPG Dihedral geometry.
Fig. S21: NMR spectroscopy describing the prevalence of trans and cis GPG isomers.
Fig. S22-S24: F(Q) fits for the isotopically substituted samples.
Fig. S25-S30: The effect of the empirical potential on the F(Q) fit.

Table S1: UPR and TPR indices.
Table S2: A comparison of the Urea Preference Indices derived from a series of Monte Carlo simulations utilising a) Lennard Jones and b) an empirical potential drawn from the scattering data.
Table S3: Molar composition of the solutions and their isotopic constituents.
Table S4-S7: Lennard Jones parameters.

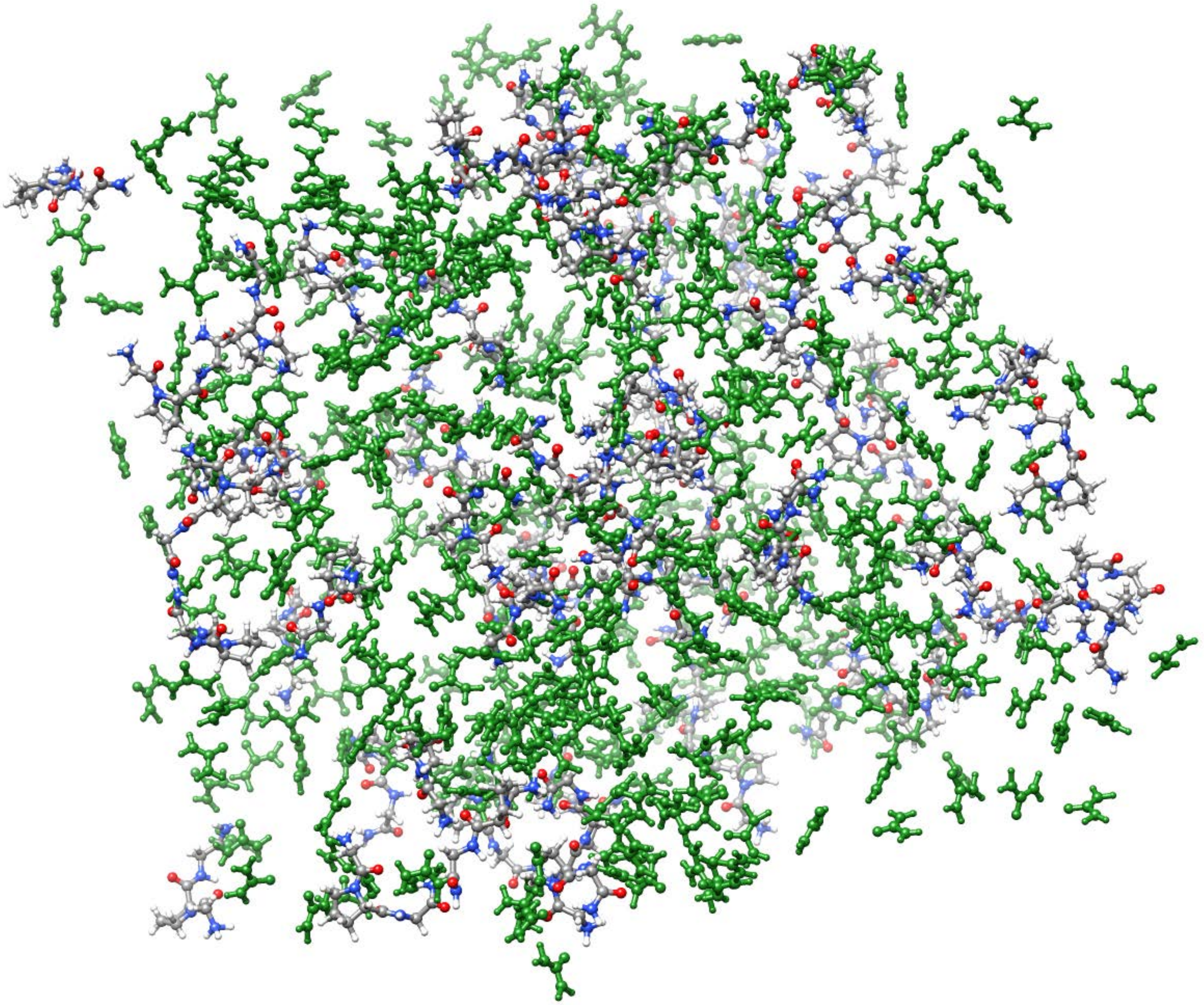

Fig. S1. The EPSR simulation box for GPG in aqueous urea imaged at the end of a simulation. The box contains 74 GPG molecules (in elemental colour), 74 chloride ions (not shown to aid visualisation), 444 urea molecules (green) and 4292 water molecules (not shown) in a cubic box of side 56.33 Å (to match the experimental atomic density, 0.106 atoms / Å^3).

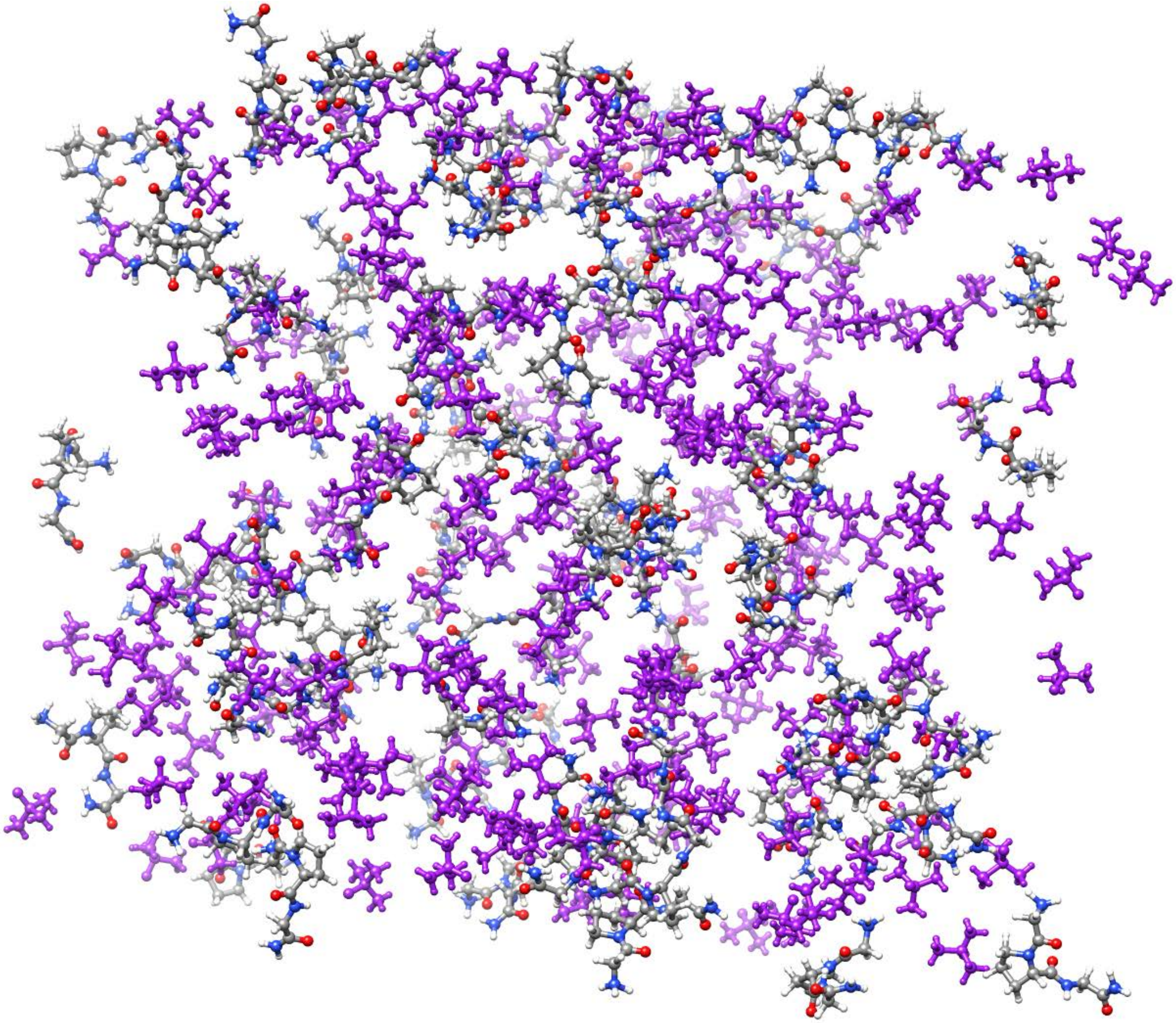

Fig. S2. The EPSR simulation box for GPG in aqueous TMAO imaged at the end of a simulation. The box contains 74 GPG molecules (in elemental colour), 74 chloride ions (not shown to aid visualisation), 222 TMAO molecules (purple) and 4292 water molecules (not shown) in a cubic box of side 56.24 Å (to match the experimental atomic density, 0.104 atoms / Å^3).

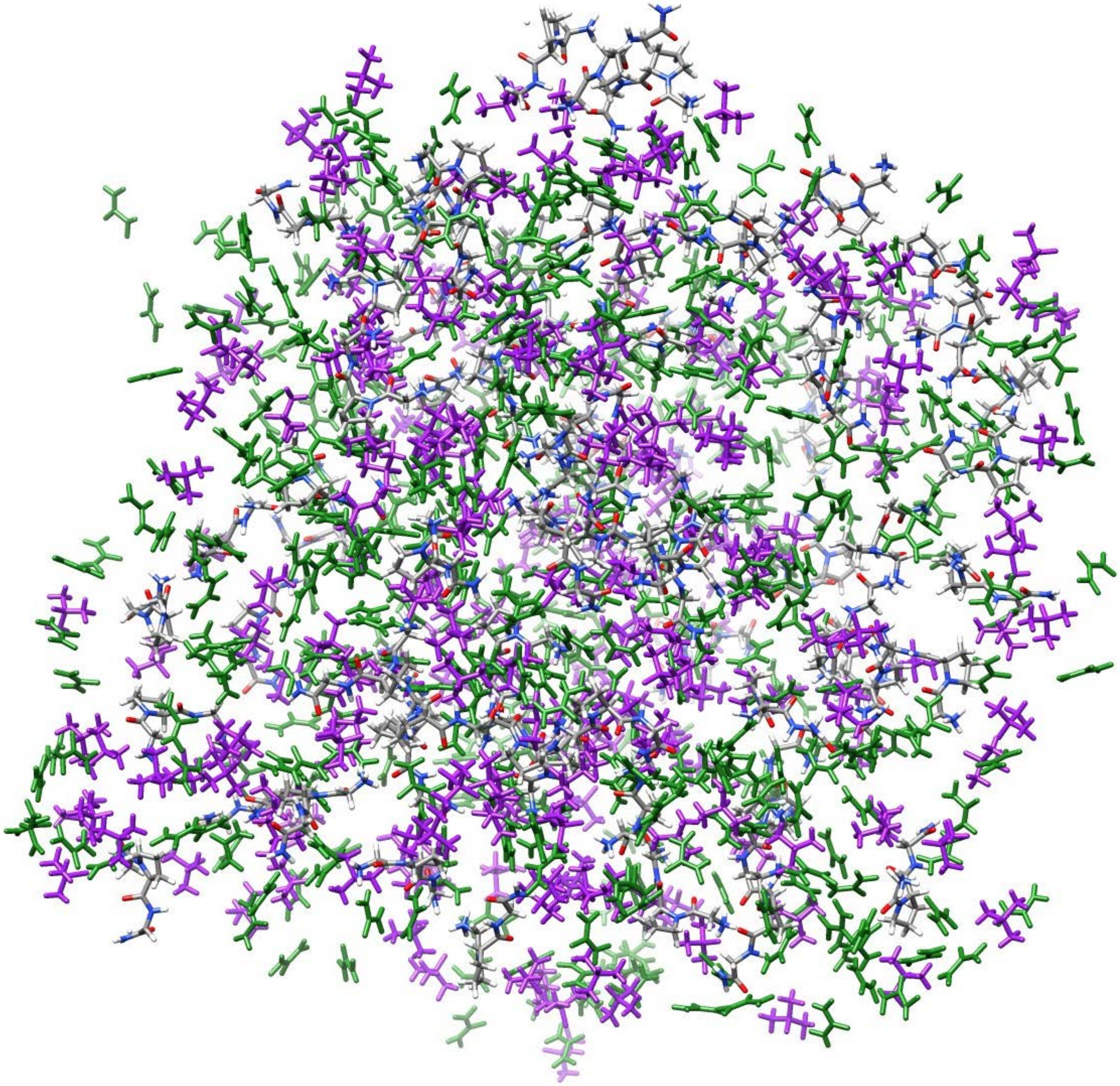

Fig. S3. The EPSR simulation box for GPG in aqueous urea-TMAO imaged at the end of a simulation. The box contains 74 GPG molecules, 74 chloride ions (not shown to aid visualisation), 222 TMAO molecules (purple), 444 urea molecules (green) and 4292 water molecules (not shown to aid visualisation) in a cubic box of side 59.25 Å generating the experimental atomic density, 0.106 atoms / Å^3).

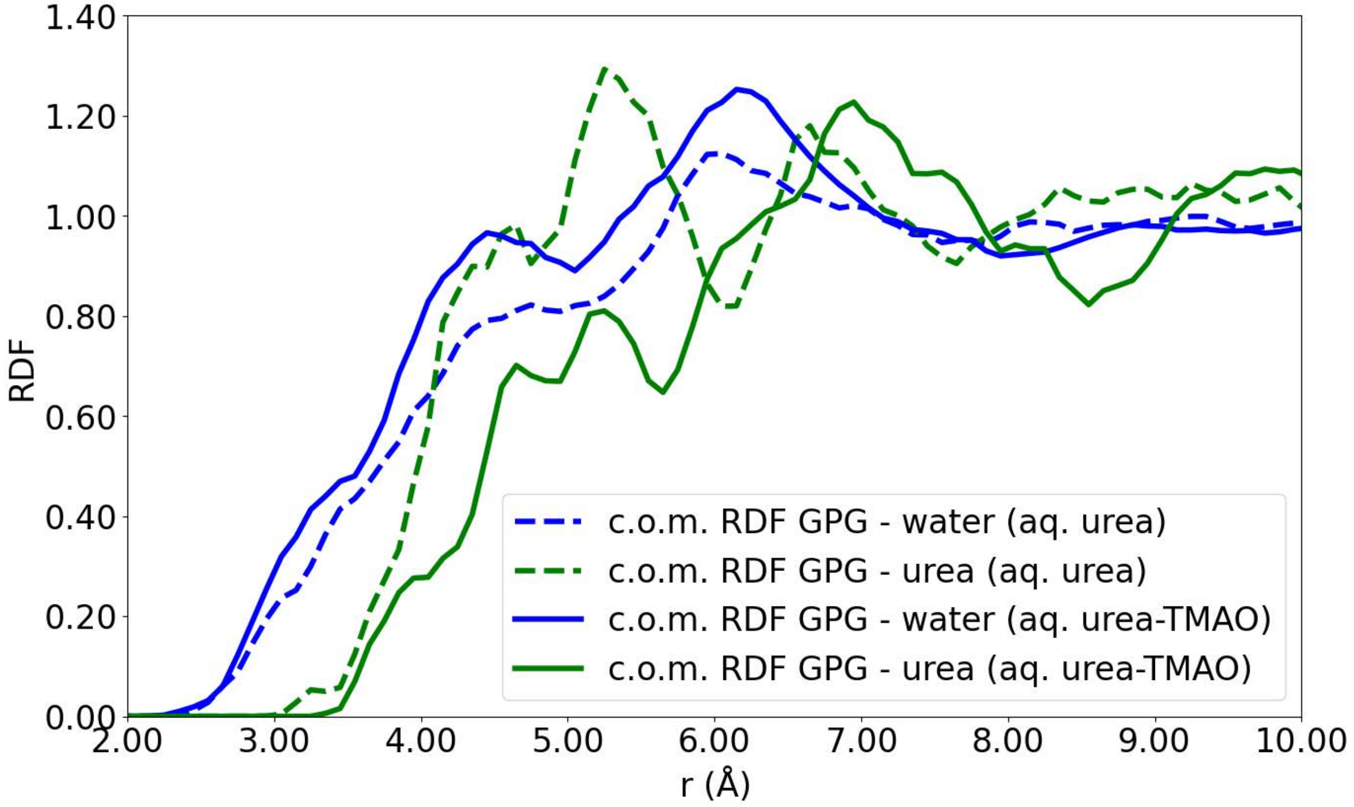

Fig. S4. The radial distribution of urea and water molecules around GPG in aqueous urea and aqueous urea and TMAO (by the centre of molecular geometry).

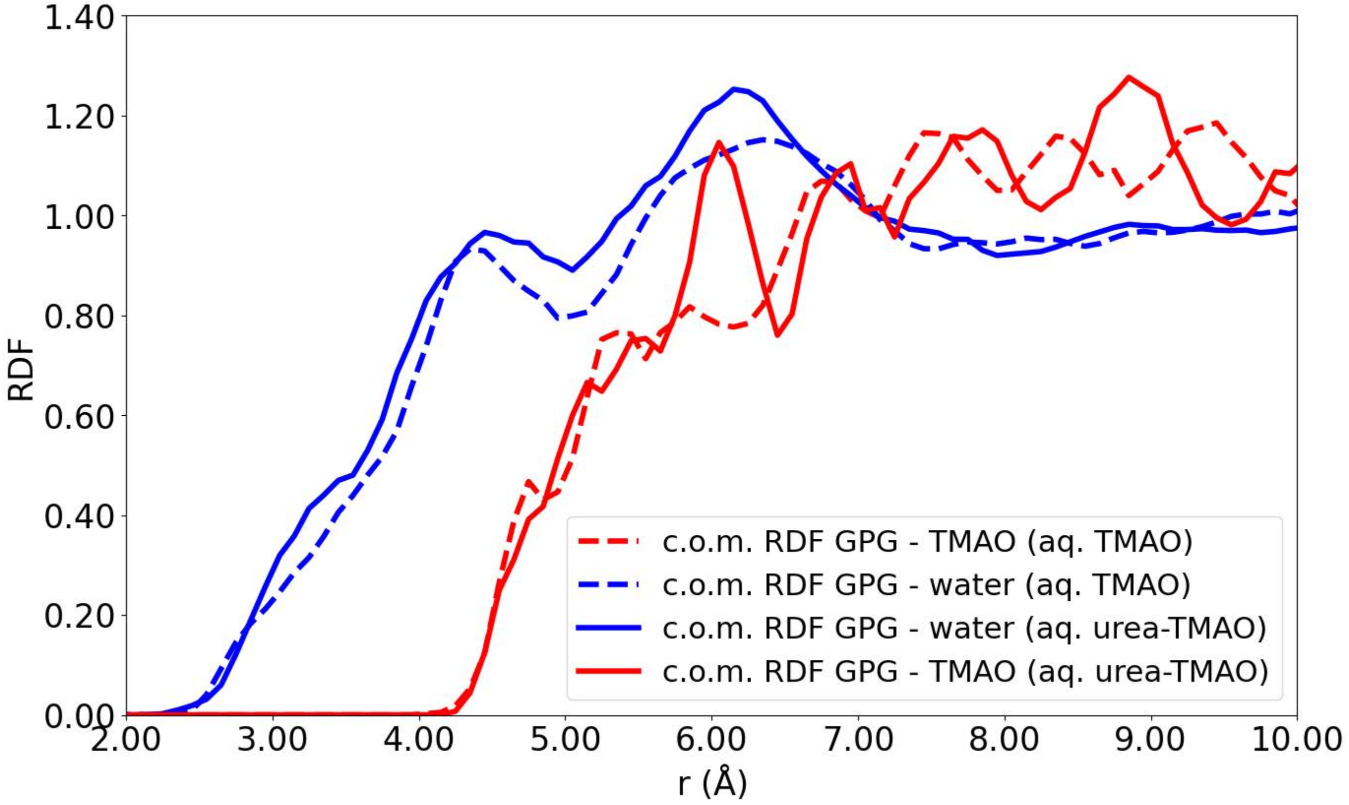

Fig. S5. The radial distribution of TMAO and water molecules around GPG in aqueous TMAO and aqueous urea and TMAO (by the centre of molecular geometry).

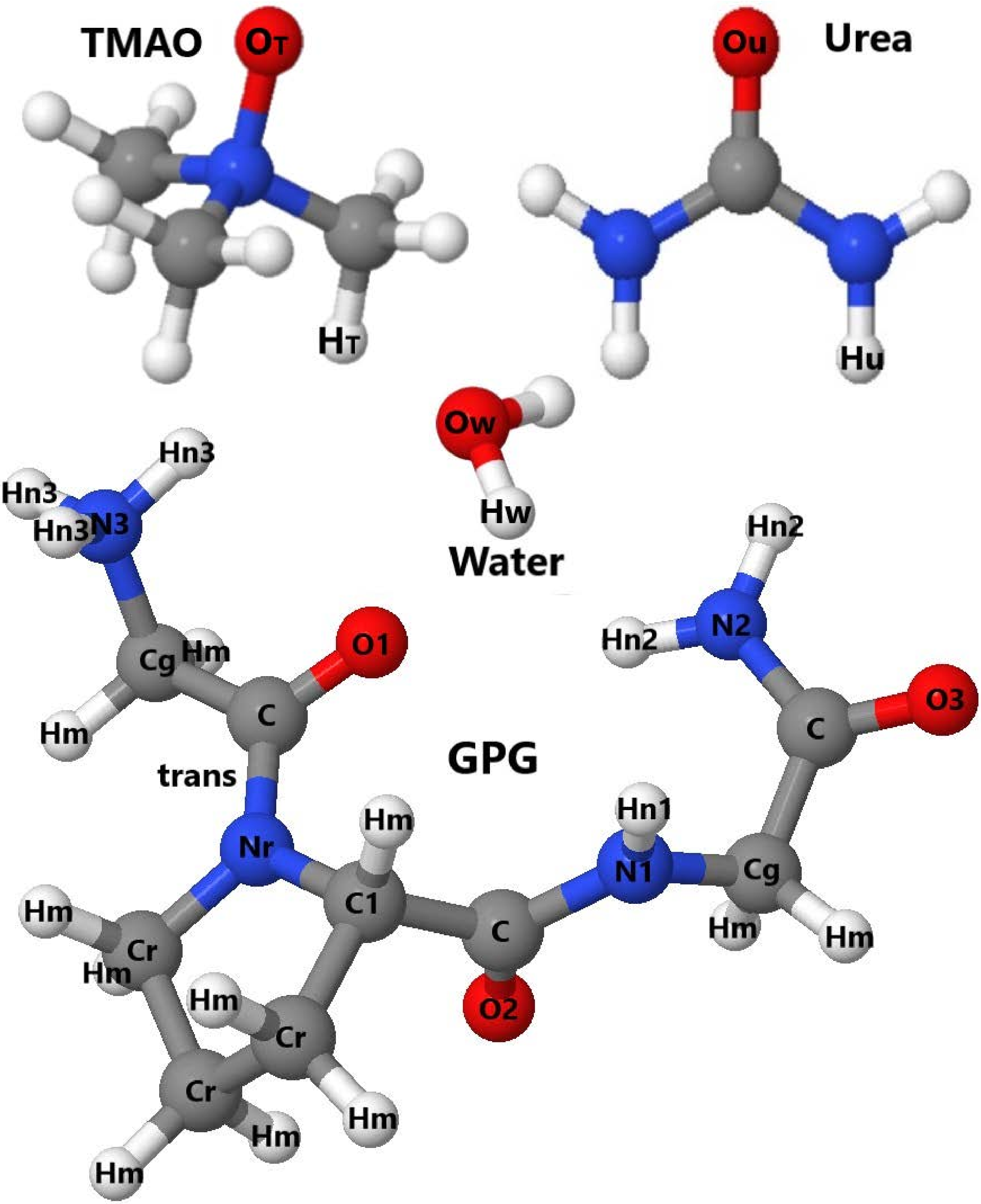

Fig. S6. The molecules and the abbreviated atom types.

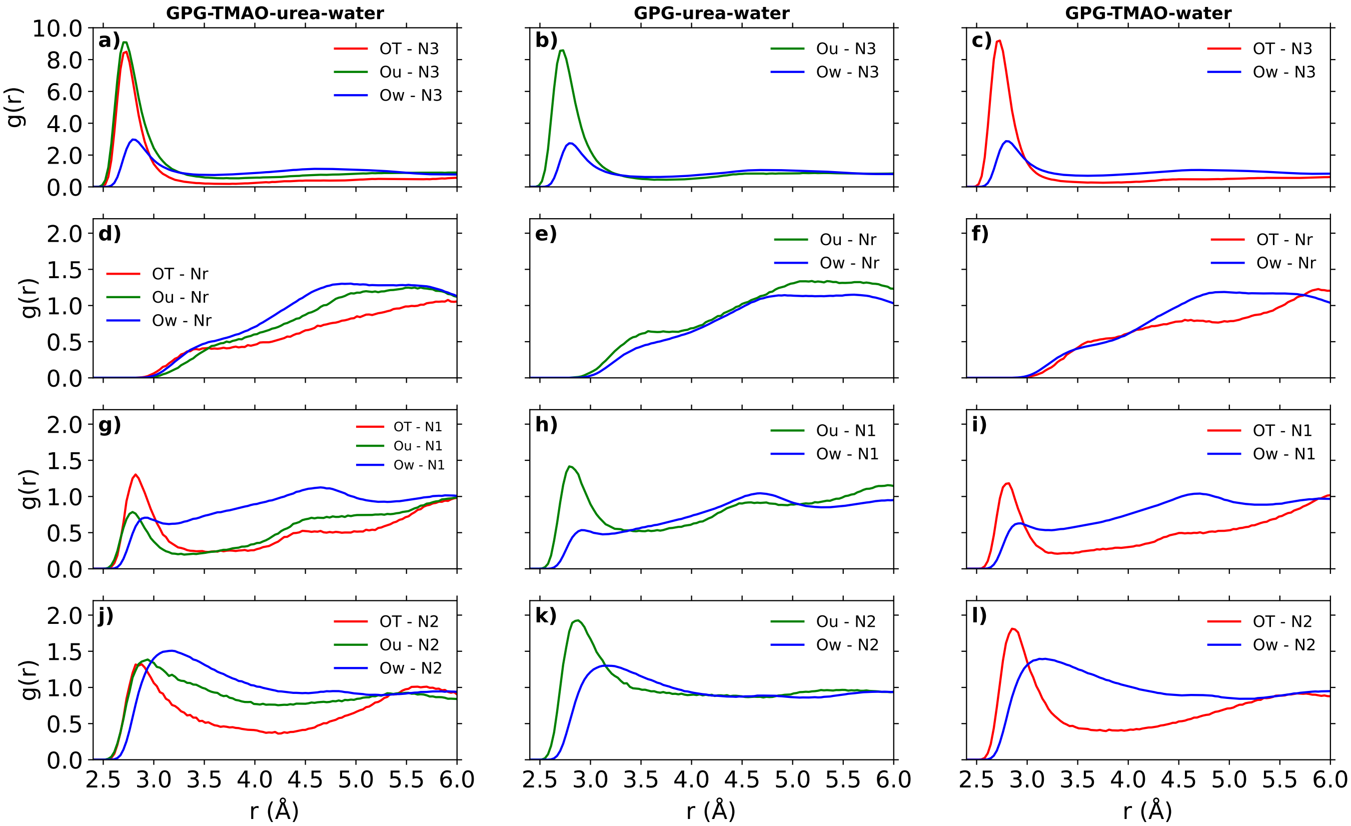

Fig. S7. The g(r) for nitrogen-peptide interactions with oxygen atoms of TMAO (OT, 'red'), urea (Ou, 'green') and water (Ow, 'blue'). Fig. S6 serves as a key to the atom types. The OT-Ni, and Ou-Ni bonds are noticeably shorter than the comparative Ow-Ni bond suggesting that urea denaturation proceeds through the urea-amide interaction. It is notable that TMAO interacts strongly with the end-nitrogen groups (N3,N2). We also see in panel (g) that in the presence of TMAO, the urea-amide interaction is very largely eroded (contrast to panel h).

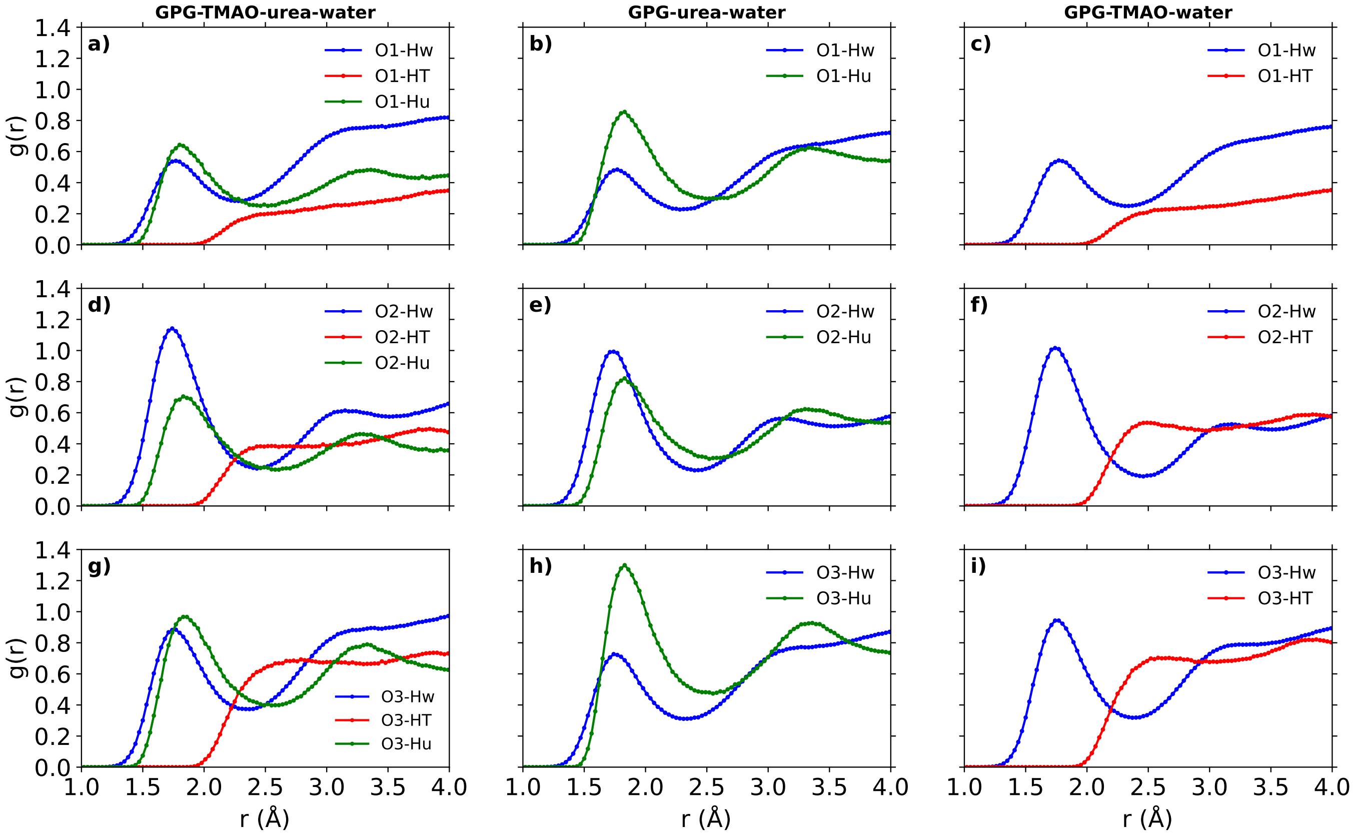

Fig. S8. The g(r) for nitrogen-peptide interactions with oxygen atoms of TMAO (OT, 'red'), urea (Ou, 'green') and water (Ow, 'blue'). Fig. S6 serves as a key to the atom types. Panels c,f,i show that TMAO is unable to interact with the oxygen atoms of the peptide (carbonyl groups). Panels b,e,h show that urea interacts strongly with the carbonyl groups in the absence of TMAO yet the Ox-Hw bond is shorter than Ox-Hu, suggesting that the urea denaturation proceeds largely through urea's interaction with the amide group. There is some variation in the height of the g$(_{Oi-Hu})$r which is likely due to the physical environment of each carbonyl group i.e., steric hindrances that we comment on in the captions to Fig. S10 and Table S2. Panels a,d, and g, show that TMAO reduces the Oi-Hu interaction (contrast to panels b,e,h)

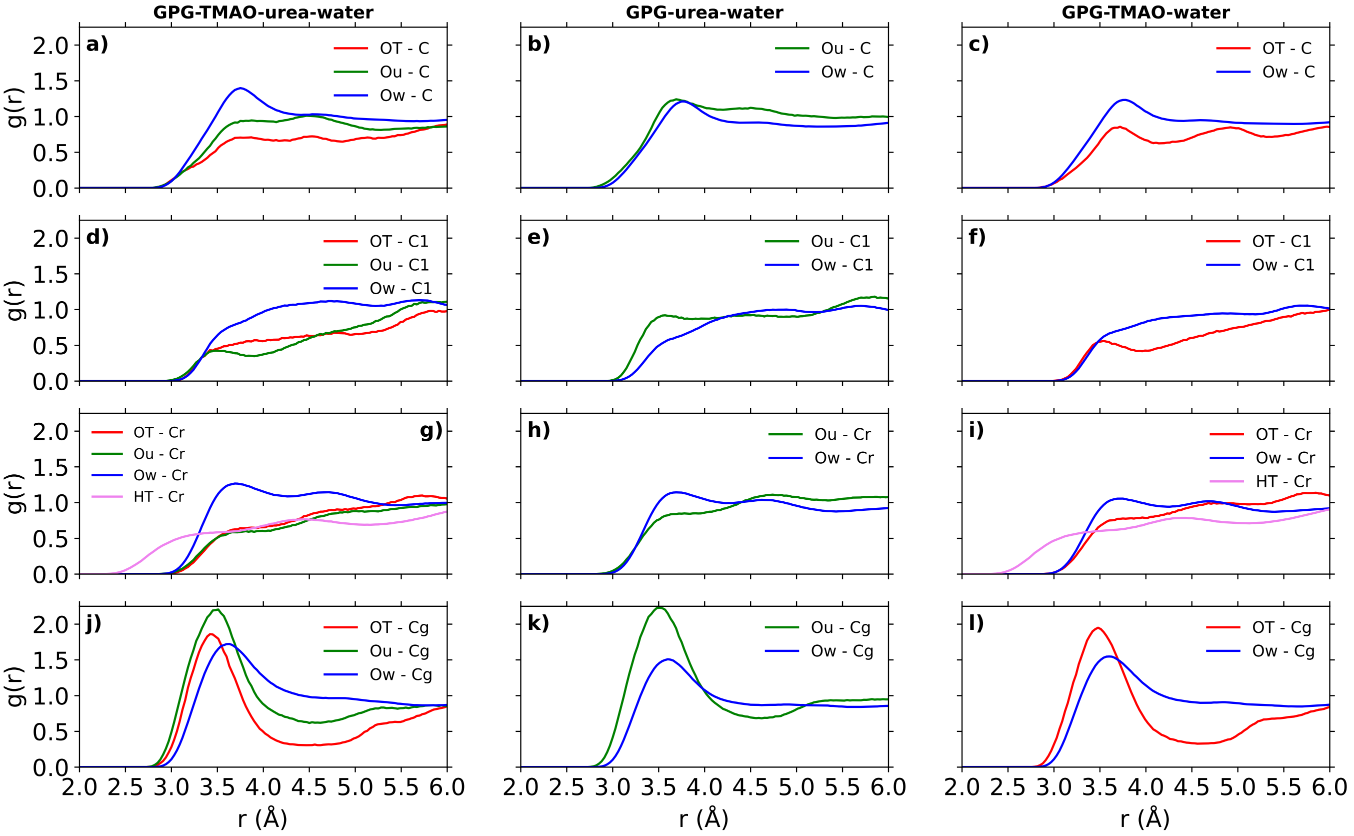

Fig. S9. The g(r) for nitrogen-peptide interactions with oxygen atoms of TMAO (OT, 'red'), urea (Ou, 'green') and water (Ow, 'blue'). Fig. S6 serves as a key to the atom types. Fig. S6 features a key to the atom types. Panels g,i also includes the H_T-Cr interaction, evidencing a weak hydrophobic interaction between TMAO and proline ring. This was the only such interaction we found on the solution systems studied. There is logic that a hydrophobic interaction should be observed here, as this is where the hydrogen atom density of the peptide is at its highest. Fig. S13 provides more detail. Panels a,d,g,j describe smaller urea features than the comparative panels b,e,h,k illustrative of the effect of TMAO on peptide-urea coordination.

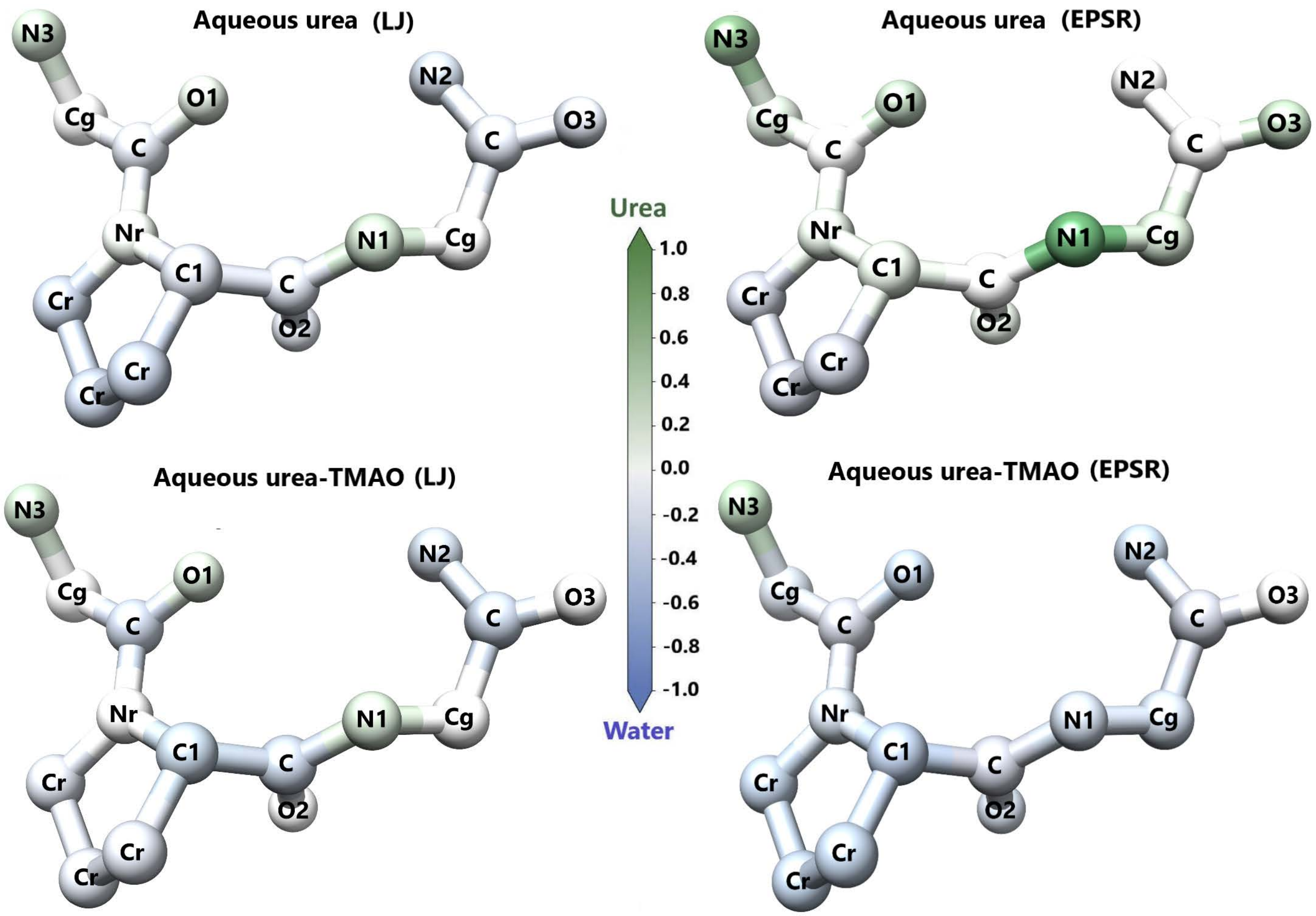

Fig. S10. A comparison of the mean relative coordination of urea and water atoms by GPG peptide atoms in a small series of Monte Carlo simulations employing a) Lennard Jones potential (LJ, left) and b) an empirical potential (EPSR, right) (1)) for GPG in aqueous urea (top) and in aqueous urea-TMAO (bottom). See Table S2 for full details. This result shows that urea's affinity for peptide atoms, in aqueous urea, can be derived from the LJ simulation alone, but in contrast the TMAO-induced depletion of urea from the tripeptide's surface, particularly at the amide atom, is consistently reproducible only in those models refined by the scattering data, i.e., with EPSR. This figure, and the underlying data complement Fig. 3 but they are not intended to replace it (the molecular trajectories of some of the simulations are smaller in extent) but the error analysis it allows (Table S2) demonstrates that the TMAO-induced urea depletion is driven by refinement to the multiple neutron scattering datasets.

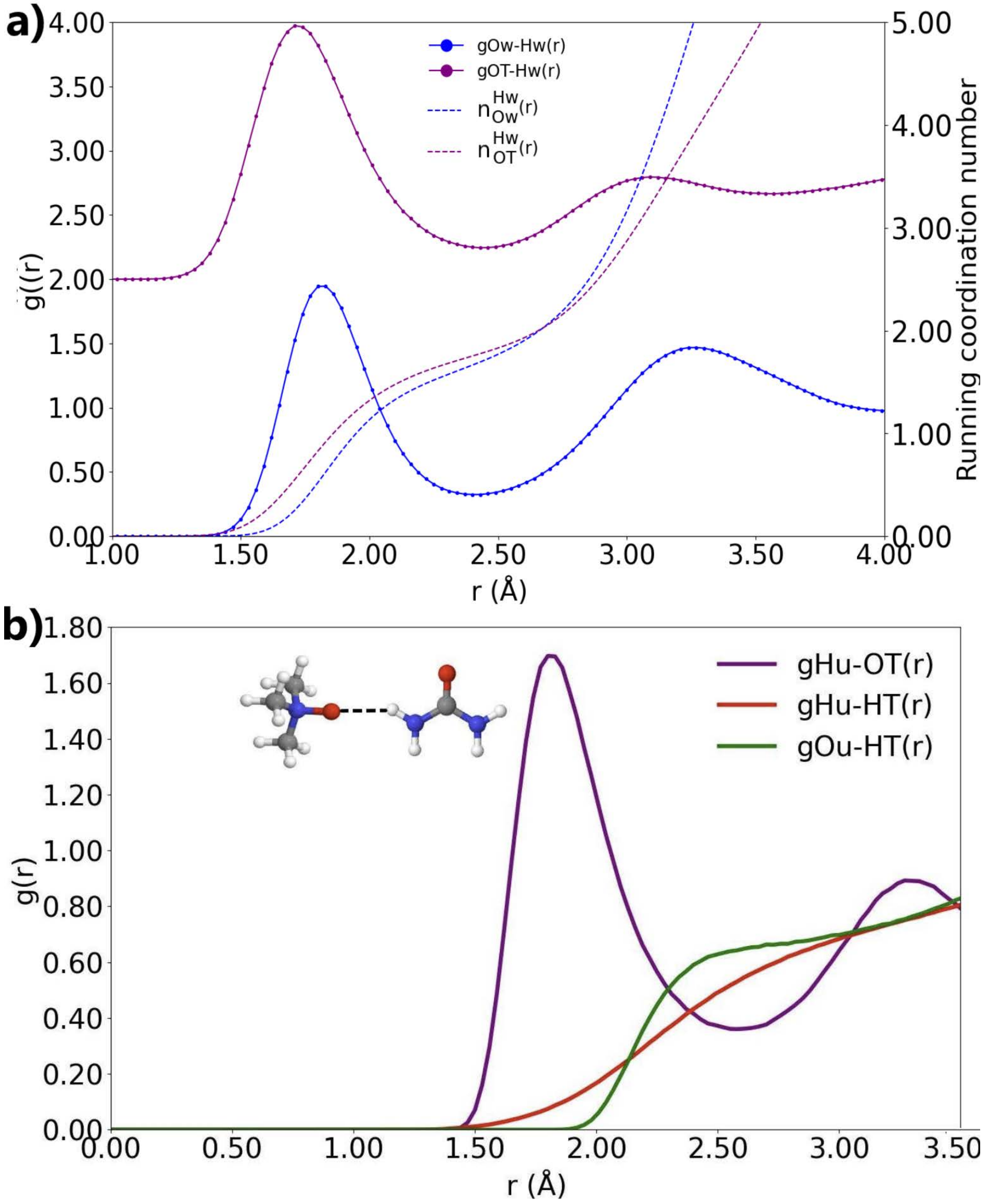

Fig. S11. a)The $g_{O_T\text{-}H_W}$(r), and $g_{O_W\text{-}H_W}$(r) in aqueous urea-TMAO with a vertical offset of 2 units. The bond length of O_T-H_U is 1.71 Å compared to 1.83 Å for O_W-H_W in aqueous urea-TMAO, an indication of the strength of the TMAO-water hydrogen bond. The dotted purple lines show that at these bond lengths, the central TMAO oxygen atom is coordinated by $\sim$ 1.80 hydrogen atoms of water, and central oxygen atoms of water are coordinated by $\sim$ 1.65 hydrogen atoms of water (in aqueous TMAO-urea). b)The possible TMAO-urea interactions. The only detectable TMAO-urea interaction is the hydrogen bond.

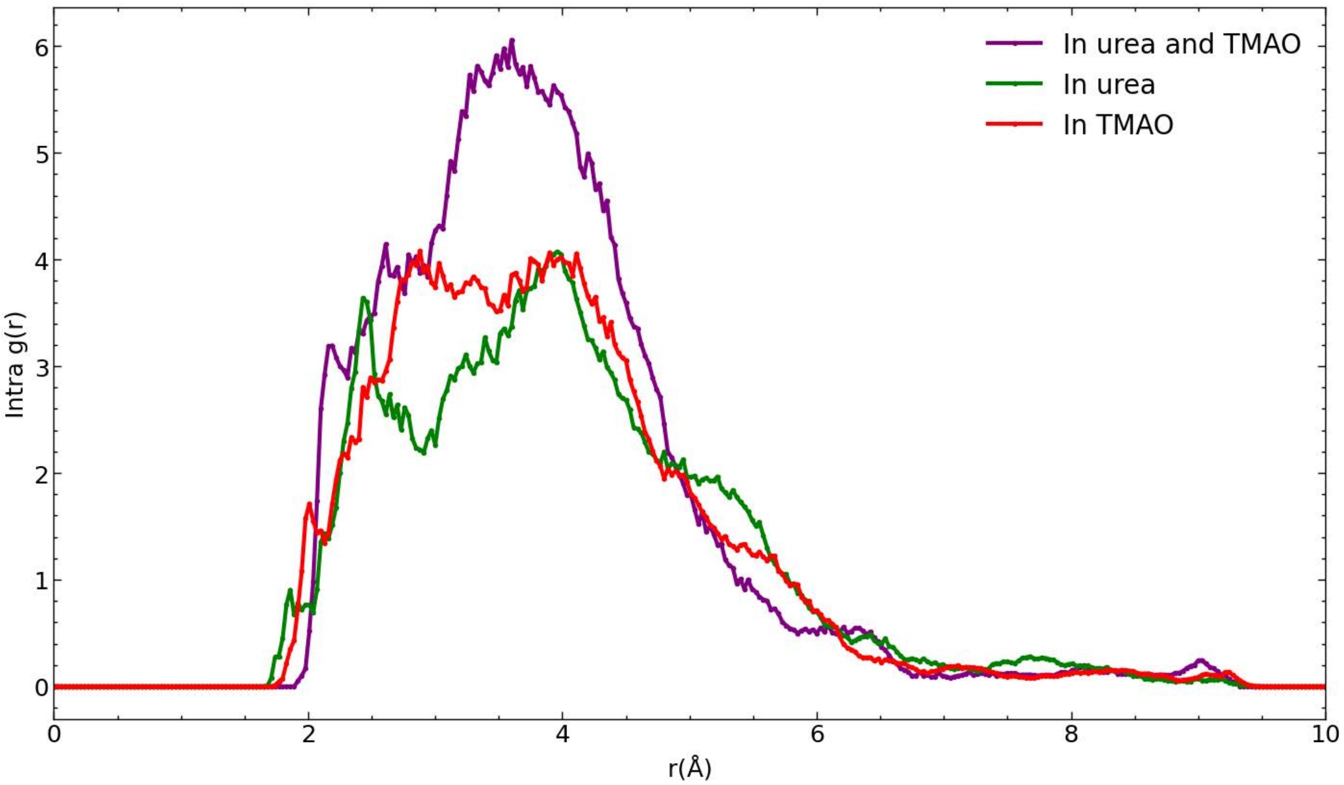

Fig. S12. The intramolecular g_{O1-Hn2}(r) in aqueous urea, aqueous TMAO, and aqueous urea-TMAO time-averaged over the course of simulations refined by the diffraction measurements. This interaction has been used by researchers (2, 3) to define the conformation of GPG in solution. The O1-Hn2 (the interaction between the first carbonyl and end amino cap) can, in some conformations, form a direct hydrogen bond. This interaction is equivalent to the interaction between the ith and $i+3^{th}$ residues that controls the flexibility of the β turn. Overall the differences in this intramolecular g(r) in aqueous urea and aqueous TMAO are small, but in the urea-TMAO solution, GPG adopts a notably more closed, or looped structure, than in the binary solutions. The g(r) in the urea-TMAO system features a small peak at $\sim$2 Å that is indicative of a weak hydrogen bond between the carbonyl and end amino-cap. This bond is shorter in aqueous TMAO, and shorter still in aqueous urea, but the small peak sizes show that these interactions in these solutions are uncommon. The dominant feature in the figure is the large diffuse peak at $\sim$3.5 Å in the urea-TMAO system. This shows that most of the GPG molecules in this solution are open loops, whereas in aqueous urea and aqueous TMAO, an increased proportion of GPG molecules adopt a more linear chain (represented by a longer O1-Hn2 distance). The large mid-range peak and weak hydrogen bond feature in the aqueous-urea system are consistent with increased O1-Hn2 interactions when urea and TMAO are complexed in solution, (freeing GPG's O1 and Hn2 sites from urea and TMAO). This behaviour is consistent with the formation of the TMAO-urea complex and suggests that it affects the conformation of the peptide in the way one would expect.

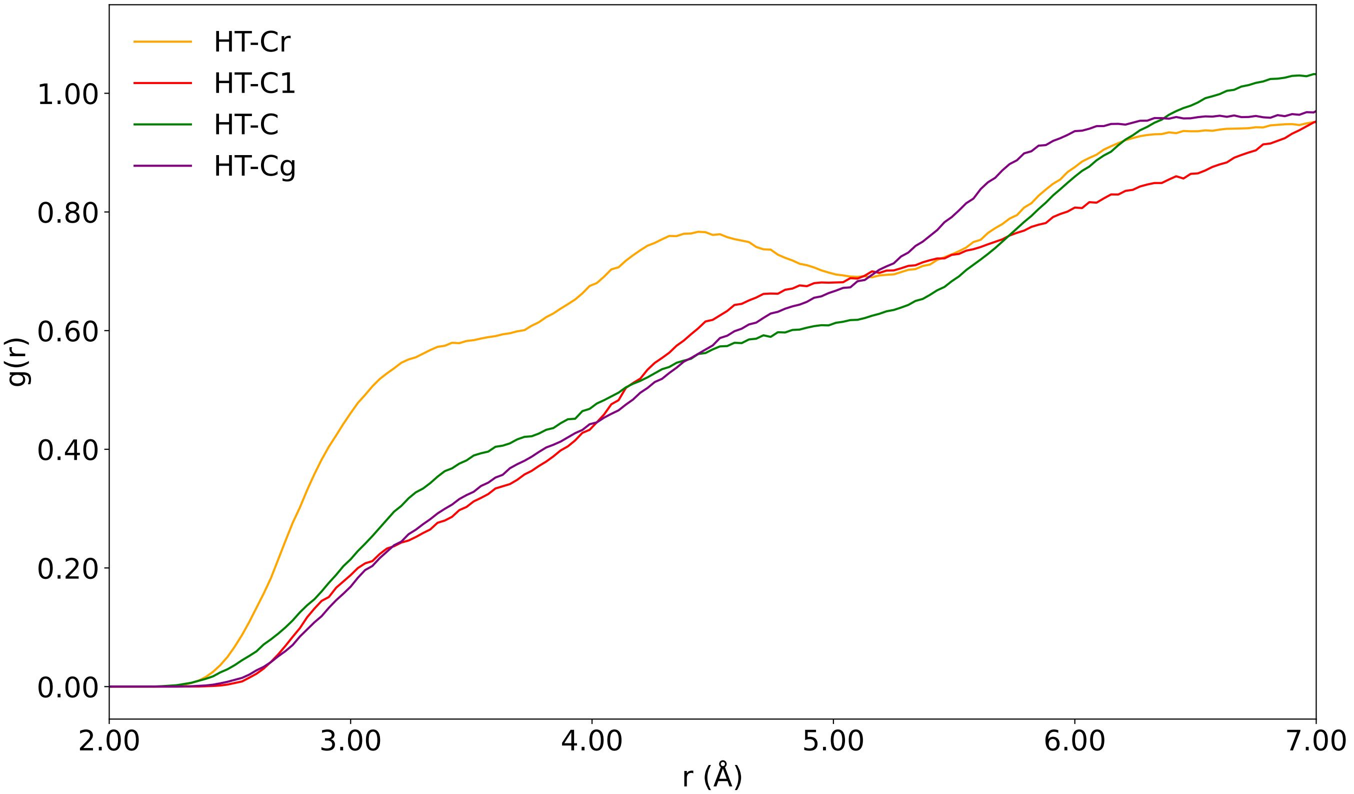

Fig. S13. The four peptide-carbon interactions with TMAO'S hydrogen atoms (H_T). The shoulder in the $g_{H_T\text{-}Cr}$(r) at $\sim$ 3 Å is indicative of a weak hydrophobic interaction between the carbon atoms of the proline ring and TMAO's hydrogen atoms (an interaction that is entirely absent from the other carbon atoms of the GPG peptide). The interaction is weak as the shoulder in the $g_{H_T\text{-}Cr}$(r) at 3 Å does not exceed 1.0 unit although if we review panels g, and i of Fig. S9 we see that around the proline ring, TMAO molecules are orientated towards the proline ring through its hydrogen atoms. This interaction was observed in the simulation box at the conclusion of an a priori simulation based solely on the atomic Lennard Jones parameters. This structure remained intact through the EPSR; therefore this feature is not driven by the diffraction datasets, but is consistent with them.

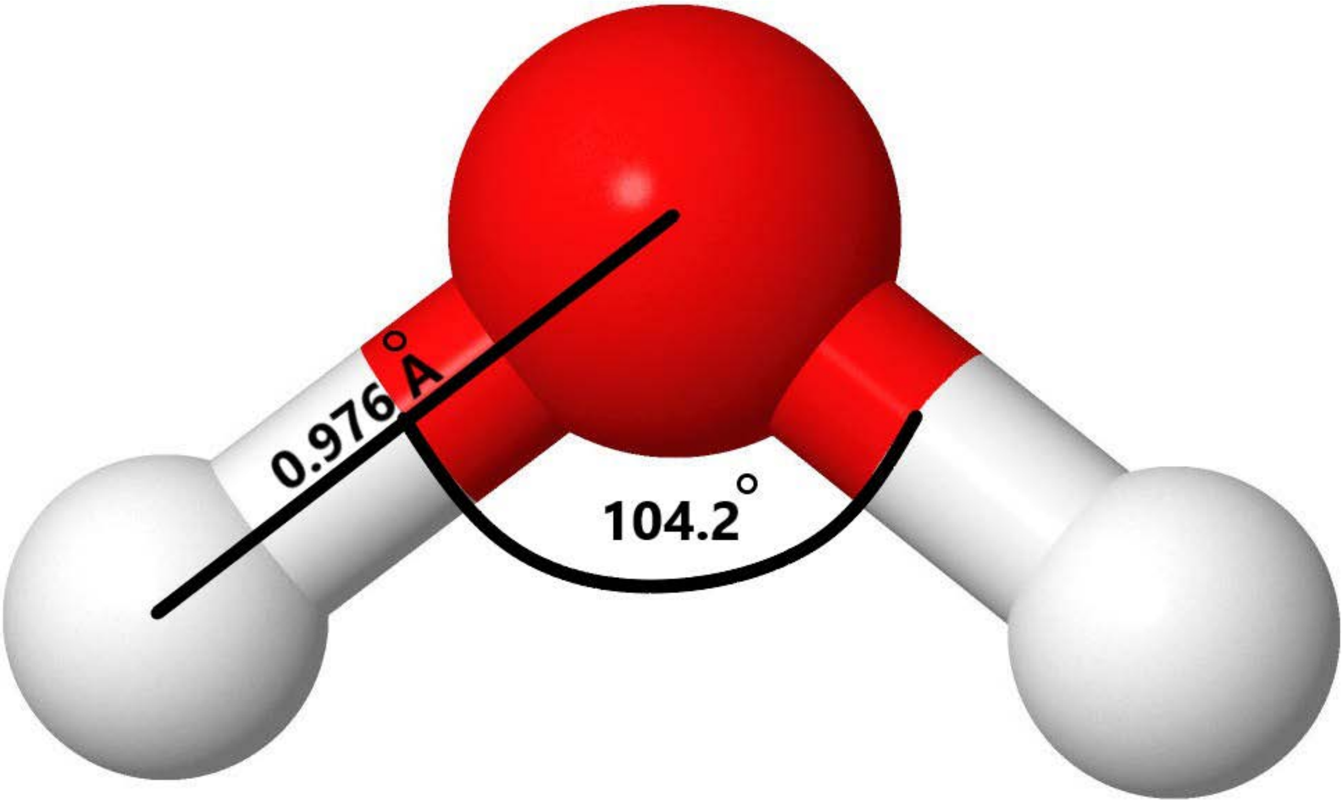

Fig. S14. The water molecule design held in EPSR

.

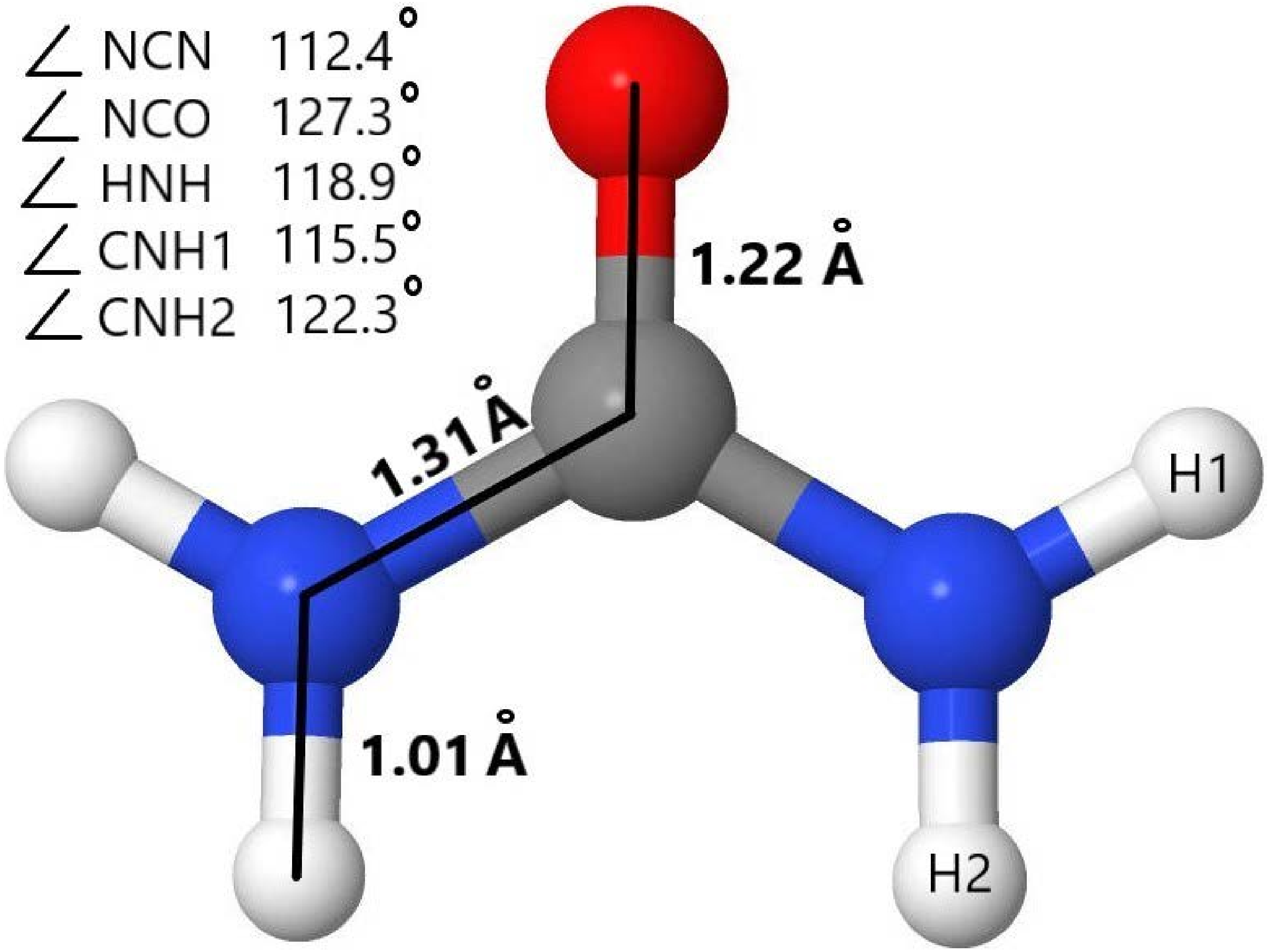

Fig. S15. The urea molecule design held in EPSR. The urea molecule was held planar by specifying the appropriate dihedral angles. The C-N bond length was subsequently changed to 1.31 Å because this produced the best fit to the diffraction measurements.

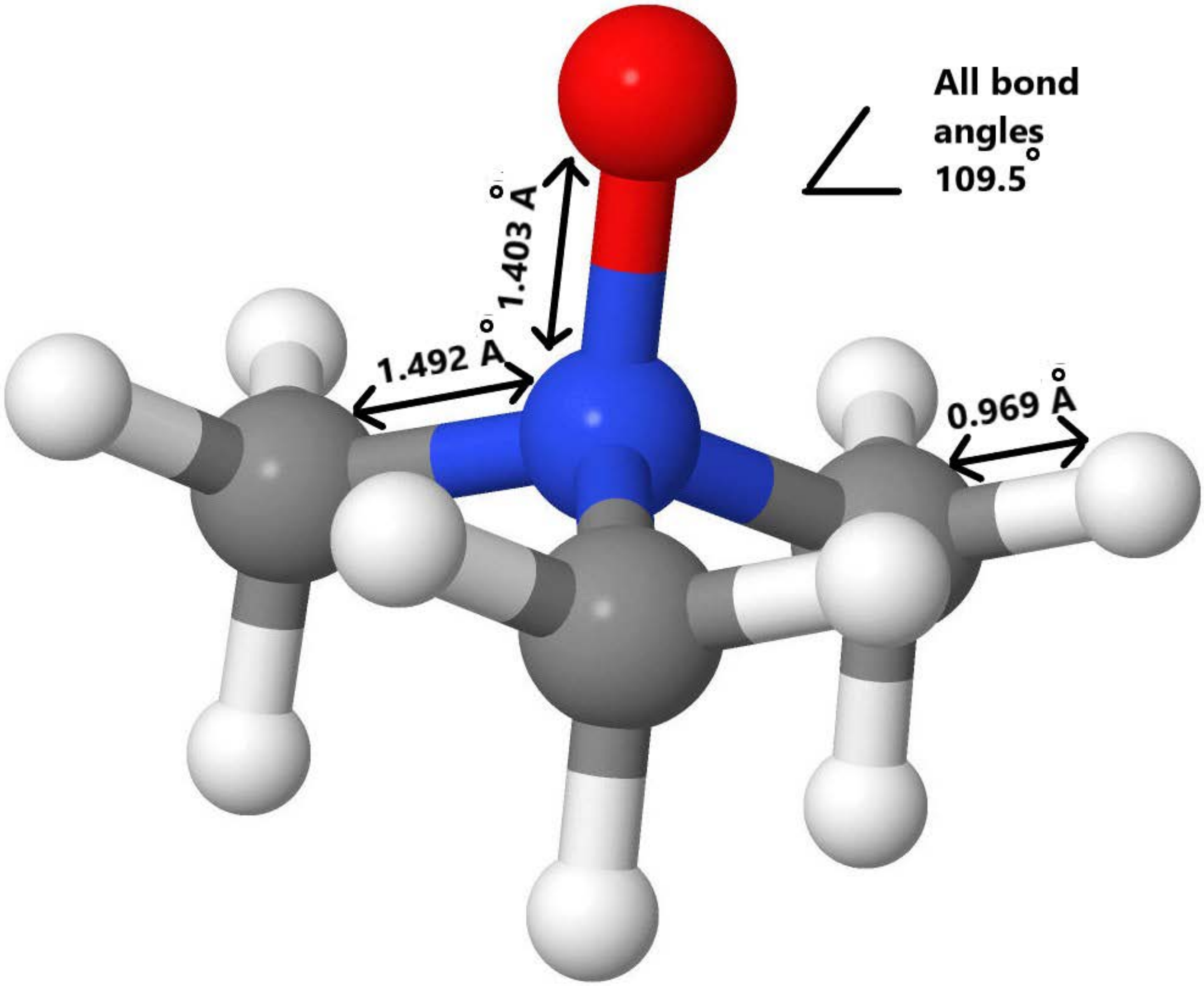

Fig. S16. The TMAO molecule design held in EPSR.

 Mazin Nasralla, Harrison Laurent, Oliver L.G. Alderman, Thomas F. Headen, and Lorna Dougan

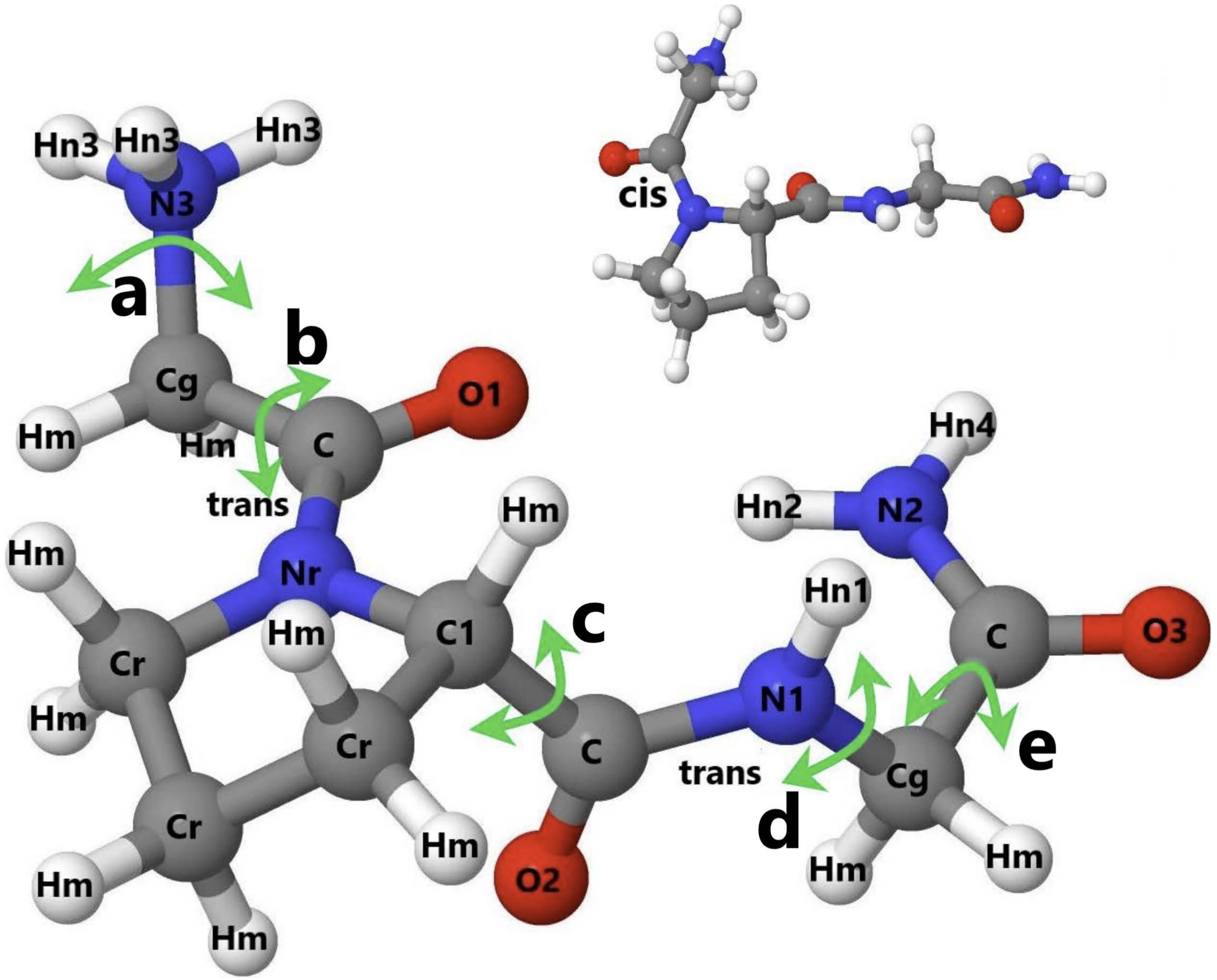

Fig. S17. The GPG ‘trans’ molecule uploaded to EPSR with the ‘cis’ conformer inset. The peptide bonds are held fixed by retaining planar dihedral angles across these bonds whilst the non-peptide, and non-proline back-bone bonds are allowed to freely rotate. The green arrows, marked ‘a’-‘e’ serve as a key to subsequent figures where the angular distribution around the rotating bonds is reported.

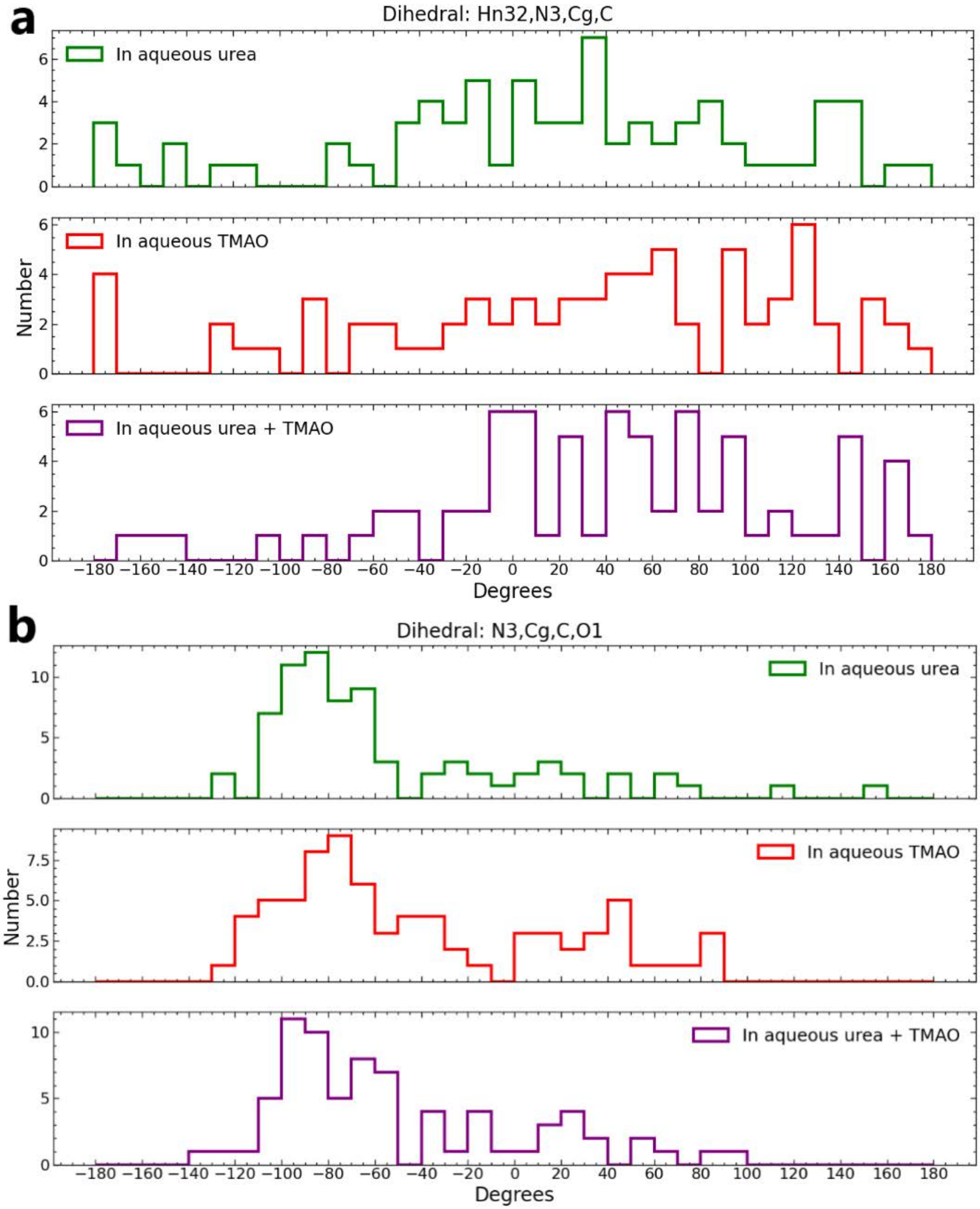

Fig. S18. A summary of the distribution of dihedral angles around the bonds marked ‘a’ and ‘b’ in Fig. S15 in aqueous urea, aqueous TMAO, and aqueous urea + TMAO based on data derived from the end of the simulation.

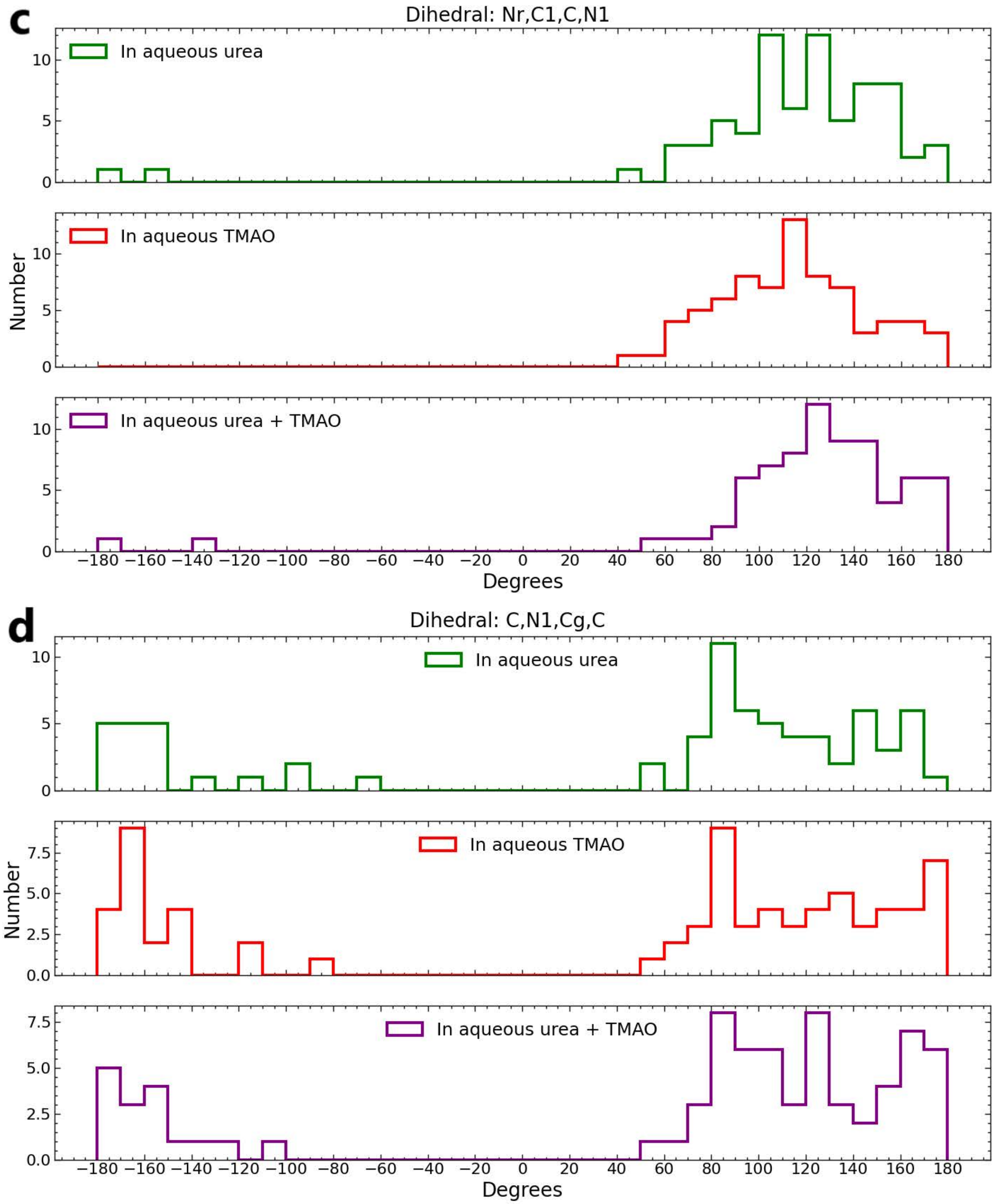

Fig. S19. A summary of the distribution of dihedral angles around the bonds marked ‘c’ and ‘d’ in Fig. S15 in aqueous urea, aqueous TMAO, and aqueous urea + TMAO based on data derived from the end of the simulation.

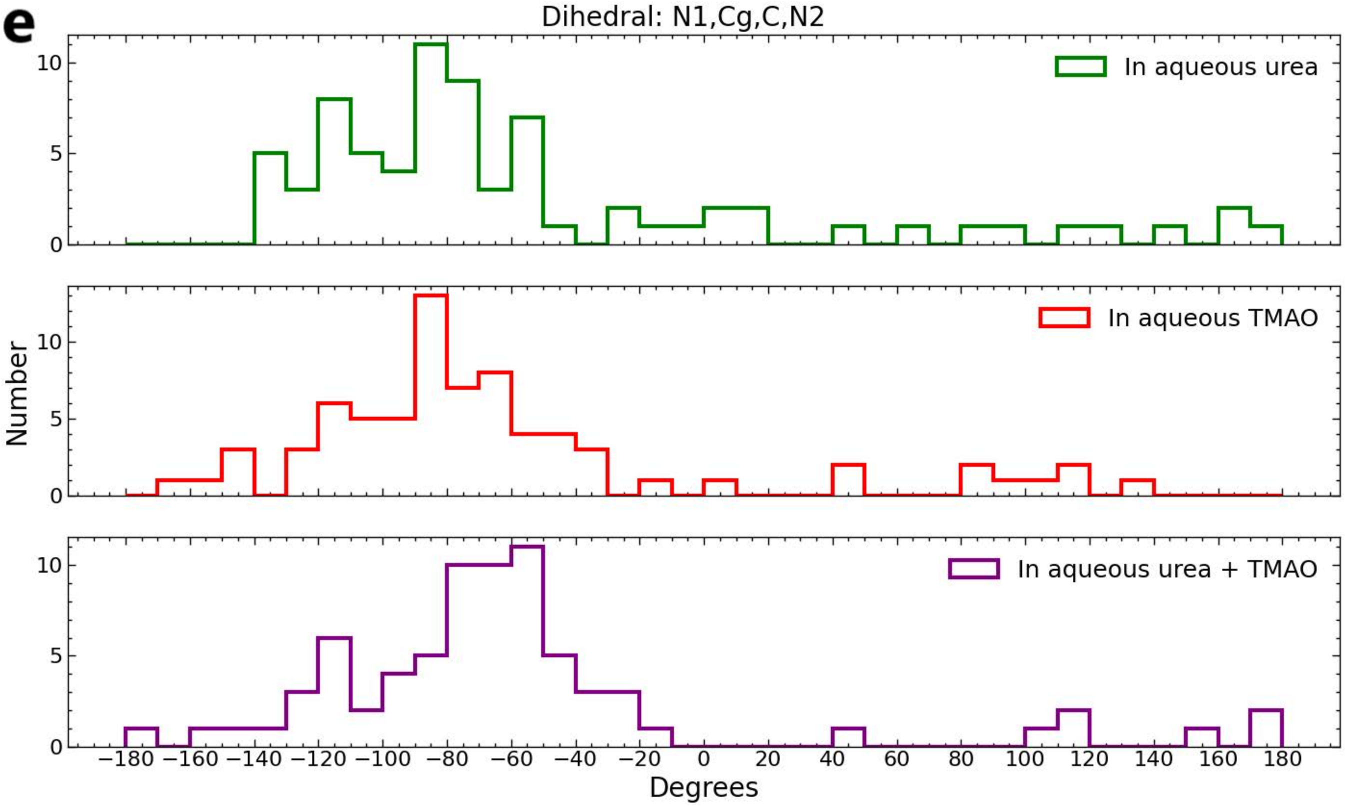

Fig. S20. A summary of the distribution of dihedral angles around bond ‘e’ in Fig. S15 in aqueous urea, aqueous TMAO, and aqueous urea + TMAO based on data derived from the end of the simulation.

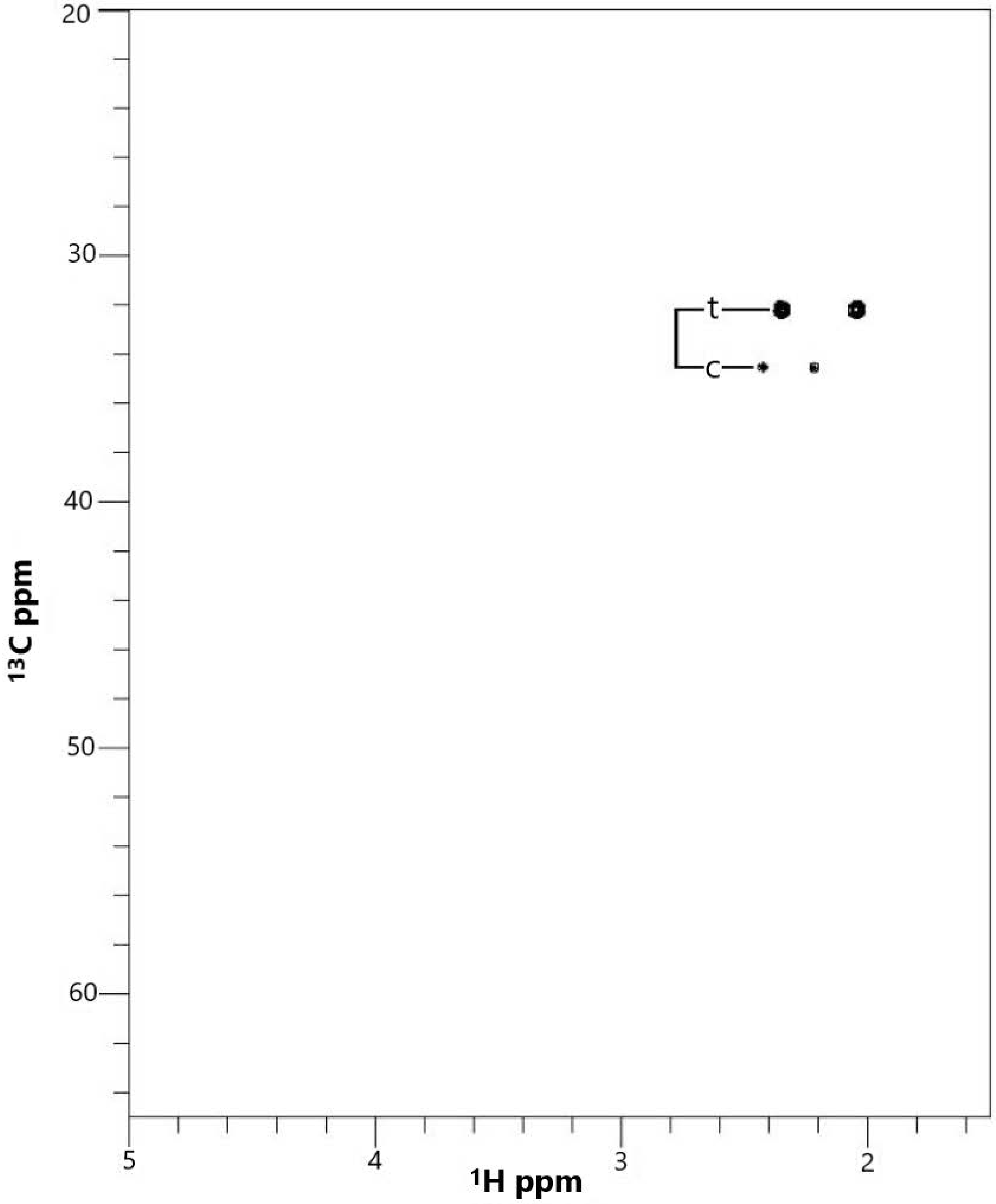

Fig. S21. HSQC NMR spectroscopy of a 30 milli-Molal GPG in 2.87 milli-Molal TMAO and 5.74 milli-Molal urea at RTP. The cis/trans ratio of GPG was derived from the proline β peak intensity ratios in water, urea, TMAO and mixed urea and TMAO solutions. The cis:trans ratio was invariant in all solutions at 12%.

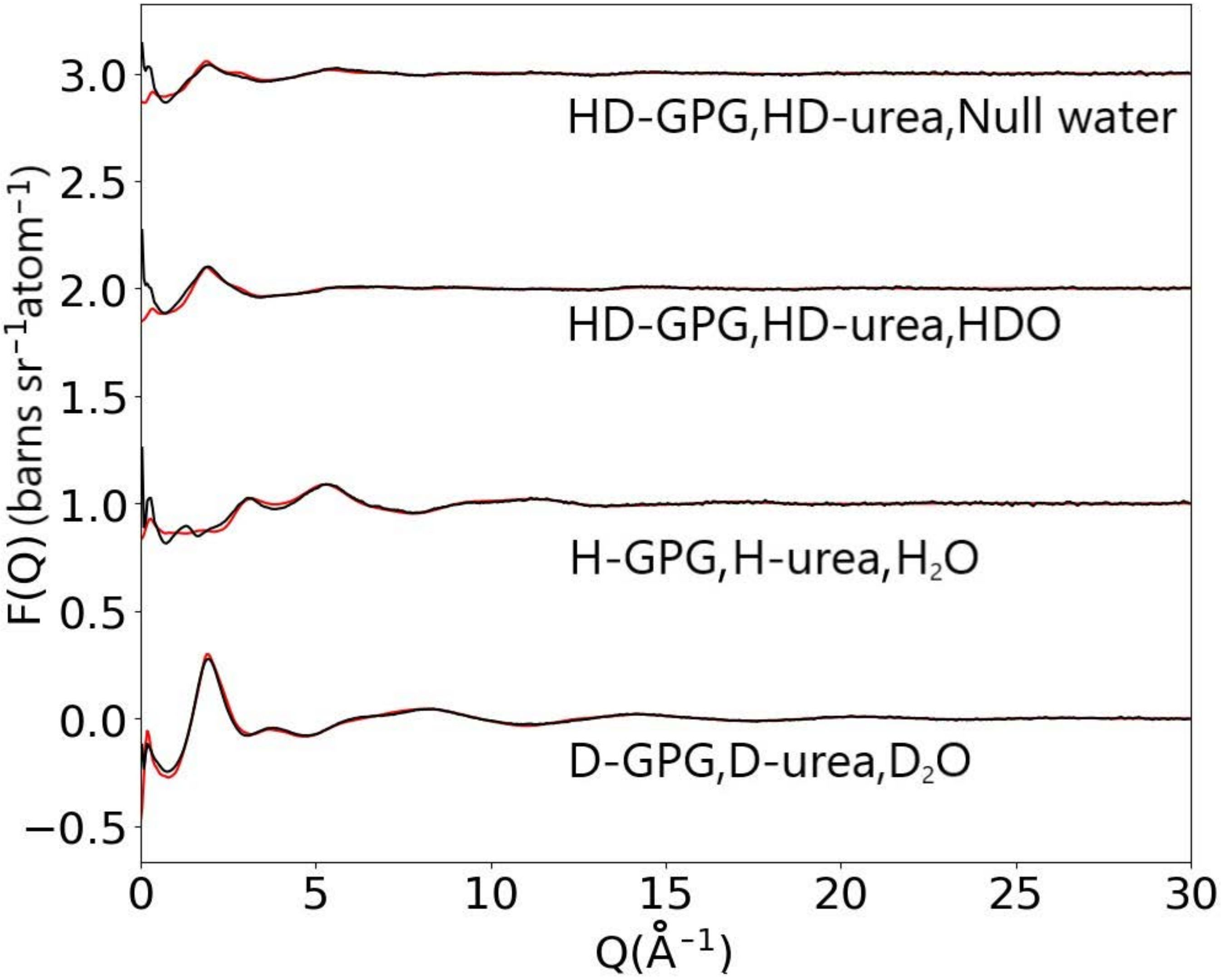

Fig. S22. F(Q) fit for GPG in aqueous urea with a vertical offset of 1 unit. The experimental and simulated F(Q) are described by black and red lines respectively. H,D,HD, Null refer to hydrogen and deuterium content of the samples. Note that in the peptide GPG, only the exchangeable hydrogen atoms (the hydrogen atoms of the nitrogen groups) were deuteriated. Null refers to a 64:36 nuclide ratio of hydrogen to deuterium.

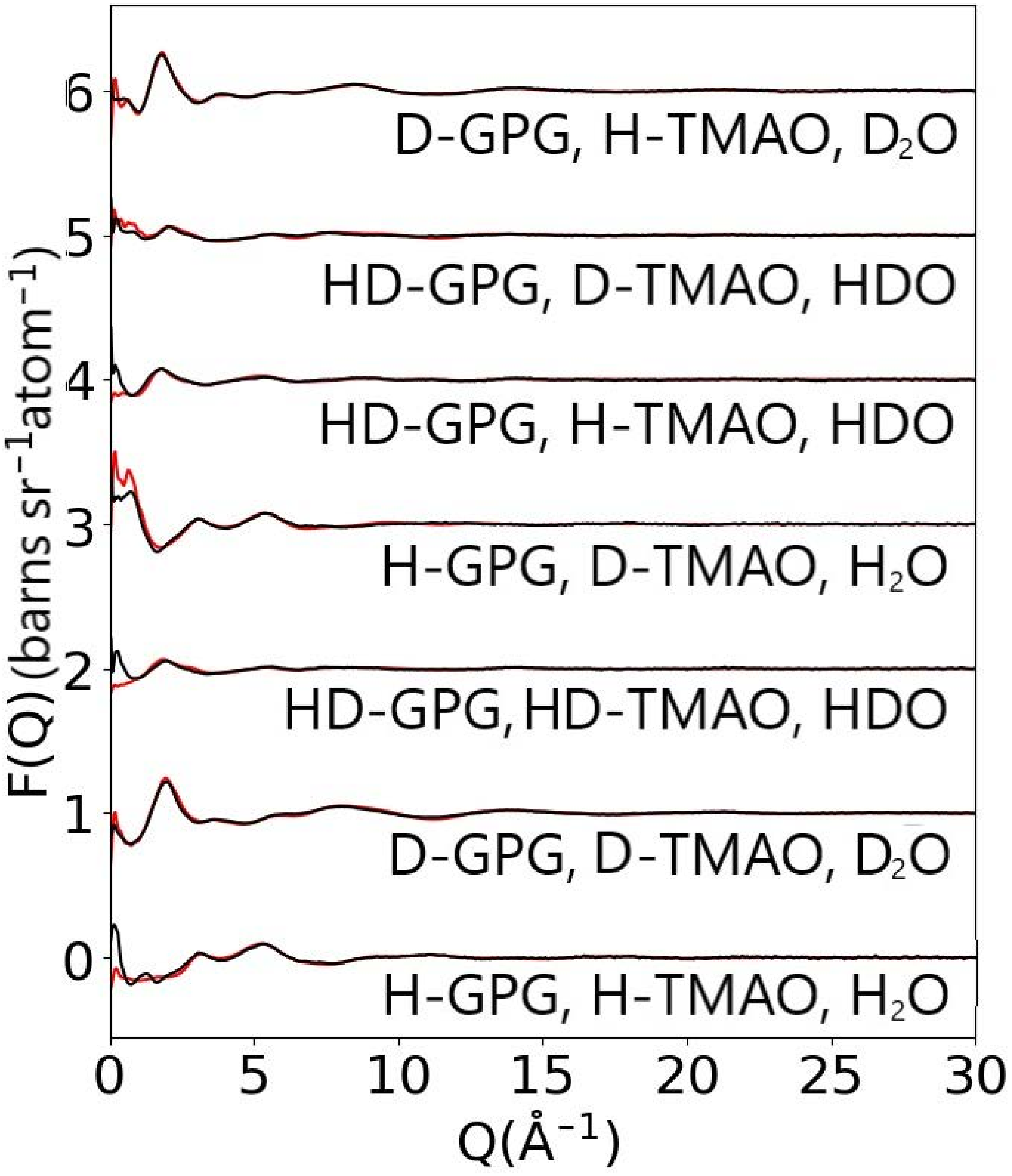

Fig. S23. The differential scattering cross section (F(Q)) for GPG in aqueous TMAO. The experimental F(Q) from NIMROD (black line) is compared to the simulated F(Q) from the EPSR simulation (red line). Each sample is offset vertically by 1.0 unit.

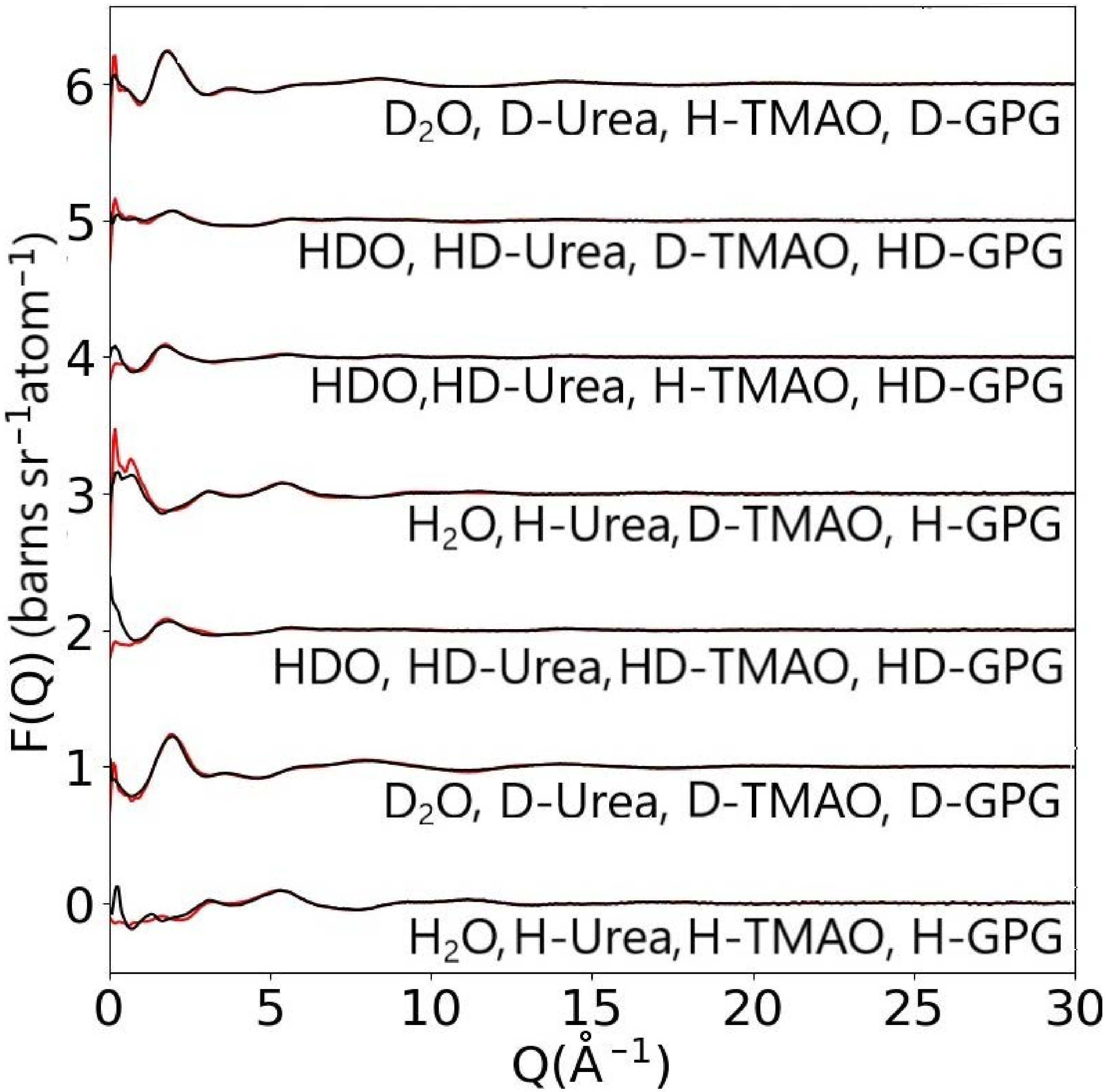

Fig. S24. The differential scattering cross section (F(Q)) for GPG in aqueous urea-TMAO. The experimental F(Q) from NIMROD (black line) is compared to the simulated F(Q) from the EPSR simulation (red line). Each sample is offset vertically by 1.0 unit.

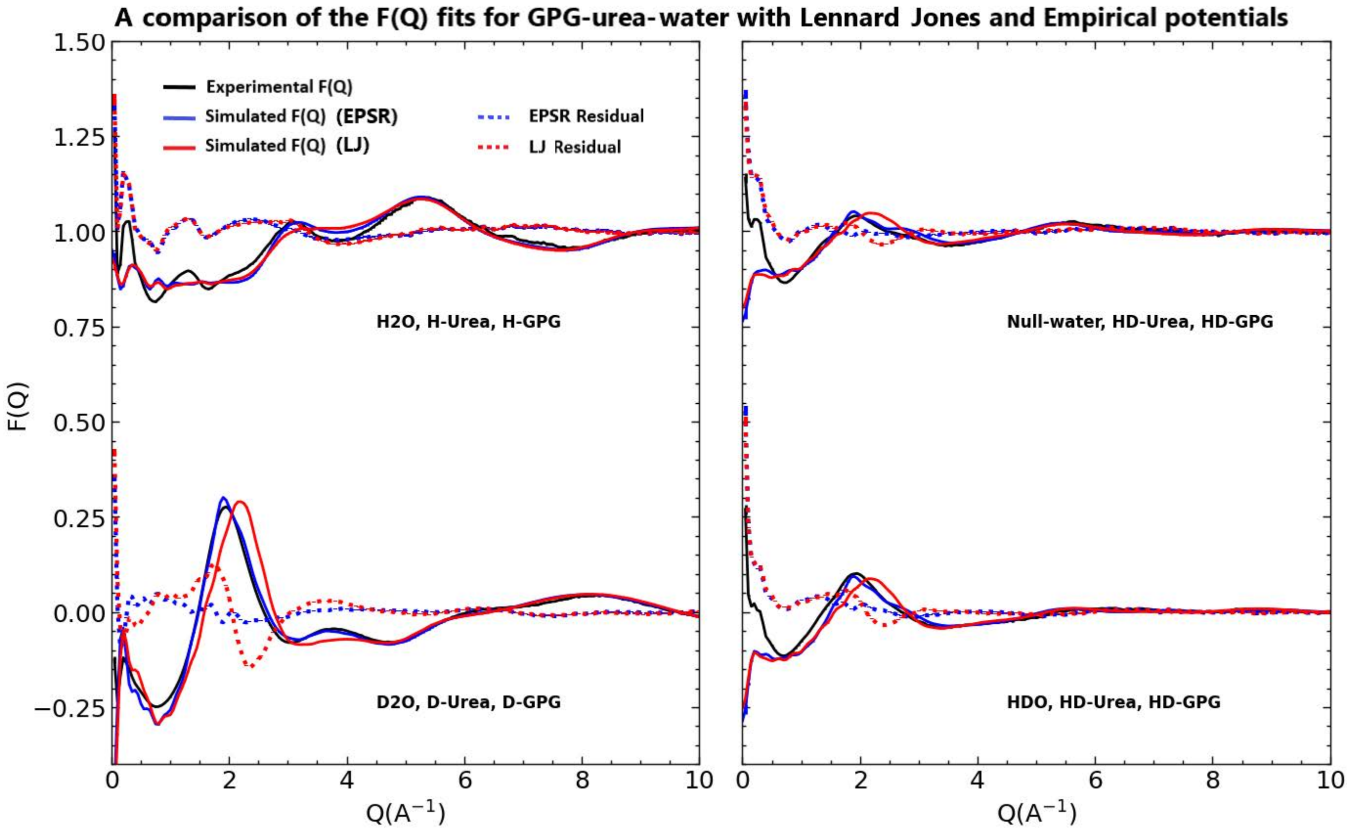

Fig. S25. A comparison of the quality of fit of the simulated D(Q) to the experimental F(Q) with reference and empirical potentials for the 4 isotopically substituted GPG-urea-water samples. The quality of fit is described here by an R Factor = $\frac{1}{M}\sum_{i}\frac{1}{n_Q(i)}\sum_{Q}[F(Q)-D(Q)]^2$, where M is the number of datasets per solution system, n_Q(i), the number of Q values found in data set i, such that a nil value describes a perfect fit. The R factor averaged over the 4 GPG samples was 0.00062 with the reference potential and 0.00047 with the empirical potential.

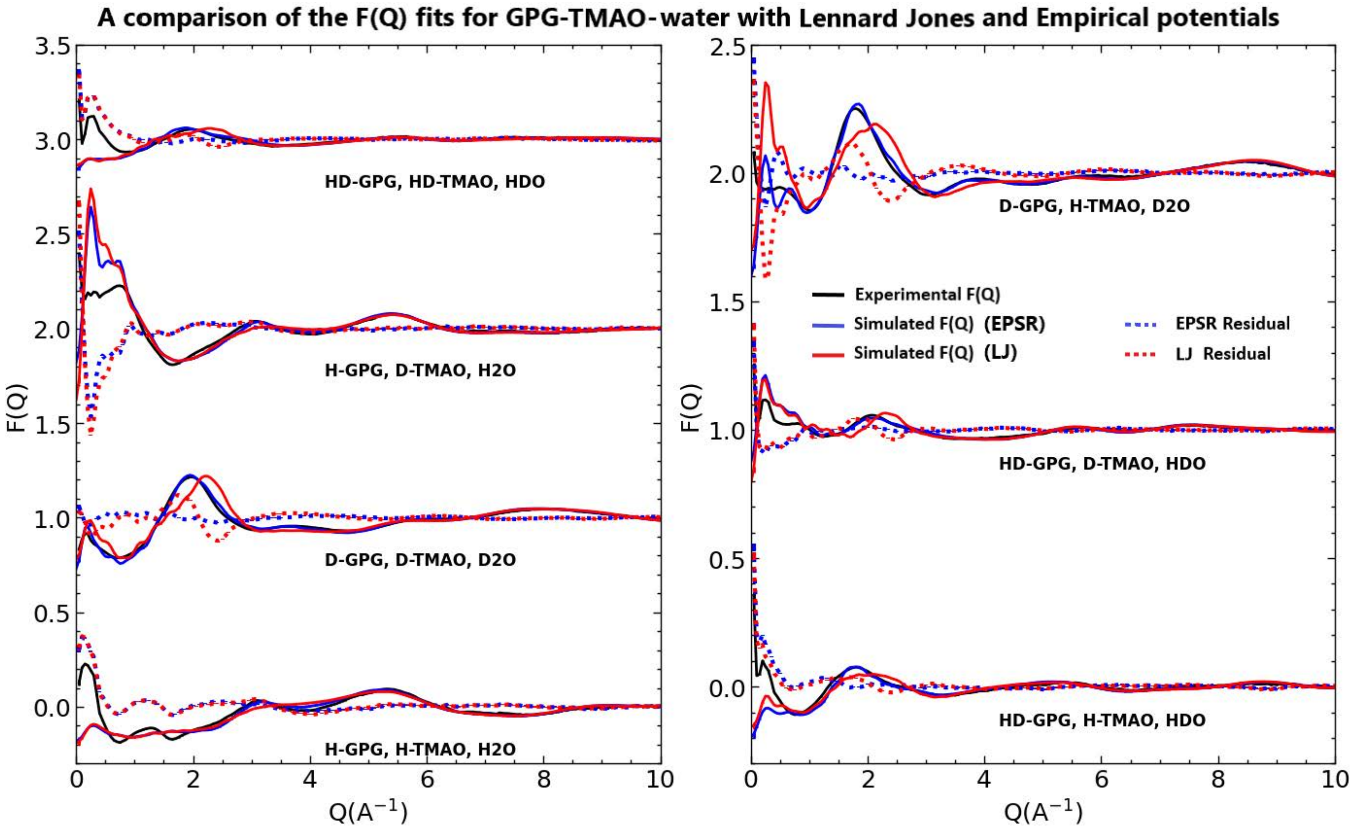

Fig. S26. A comparison of the quality of fit of the simulated D(Q) to the experimental F(Q) with reference and empirical potentials for the 7 isotopically substituted GPG-TMAO-water samples. The quality of fit is described here by an R Factor = $\frac{1}{M}\sum_i \frac{1}{n_Q(i)}\sum_Q [F(Q)-D(Q)]^2$, where M is the number of datasets per solution system, n_Q(i), the number of Q values found in data set i, such that a nil value describes a perfect fit. The R factor averaged over the 7 samples was 0.00116 with the reference potential and 0.00077 with the empirical potential.

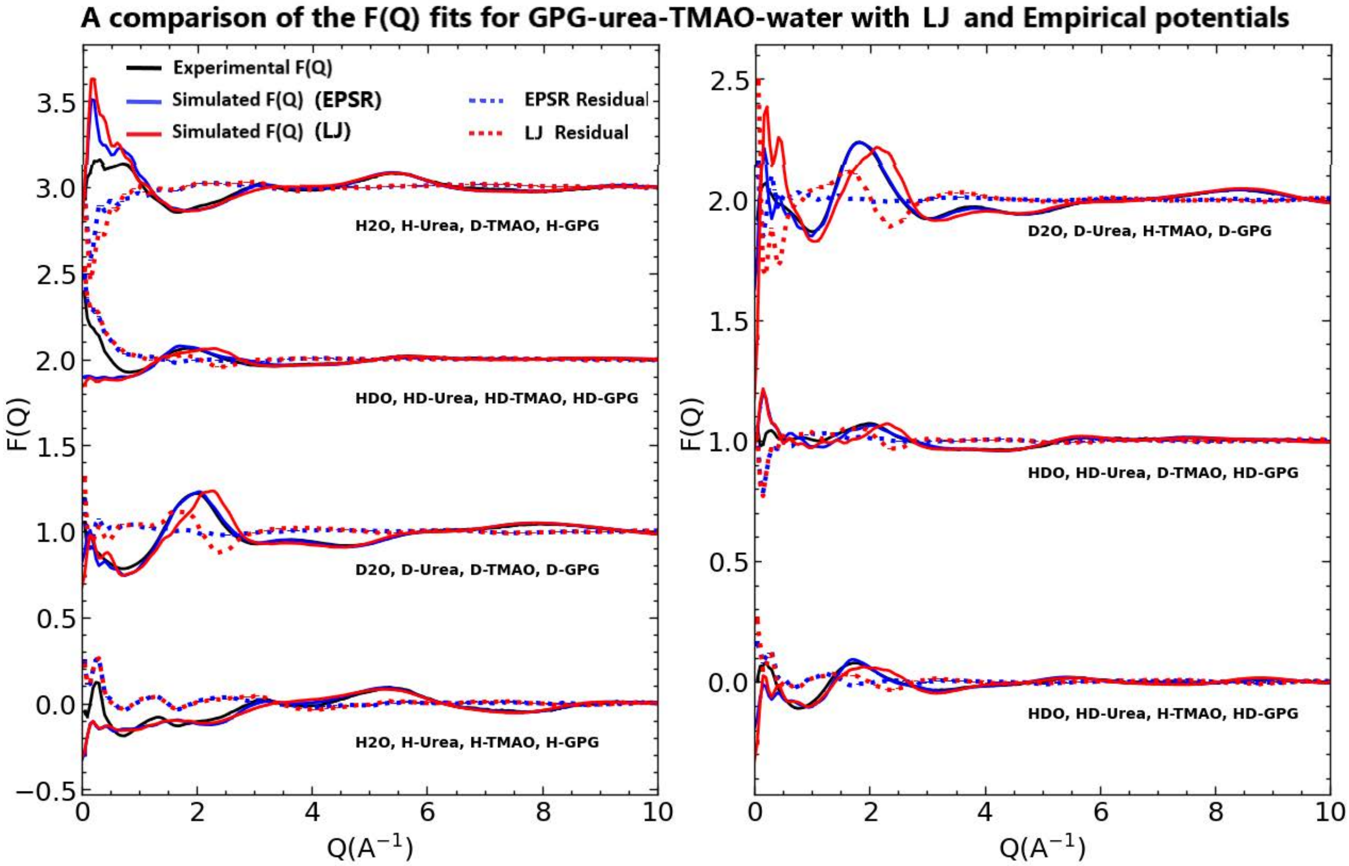

Fig. S27. A comparison of the quality of fit of the simulated D(Q) to the experimental F(Q) with reference and empirical potentials for the 7 isotopically substituted GPG-urea-TMAO-water samples. The quality of fit is described here by an R Factor = $\frac{1}{M}\sum_{i}\frac{1}{n_Q(i)}\sum_{Q}[F(Q)-D(Q)]^2$, where M is the number of datasets per solution system, n_Q(i), the number of Q values found in data set i, such that a nil value describes a perfect fit. The R factor averaged over the 7 samples was 0.00105 with the reference potential and 0.00062 with the empirical potential.

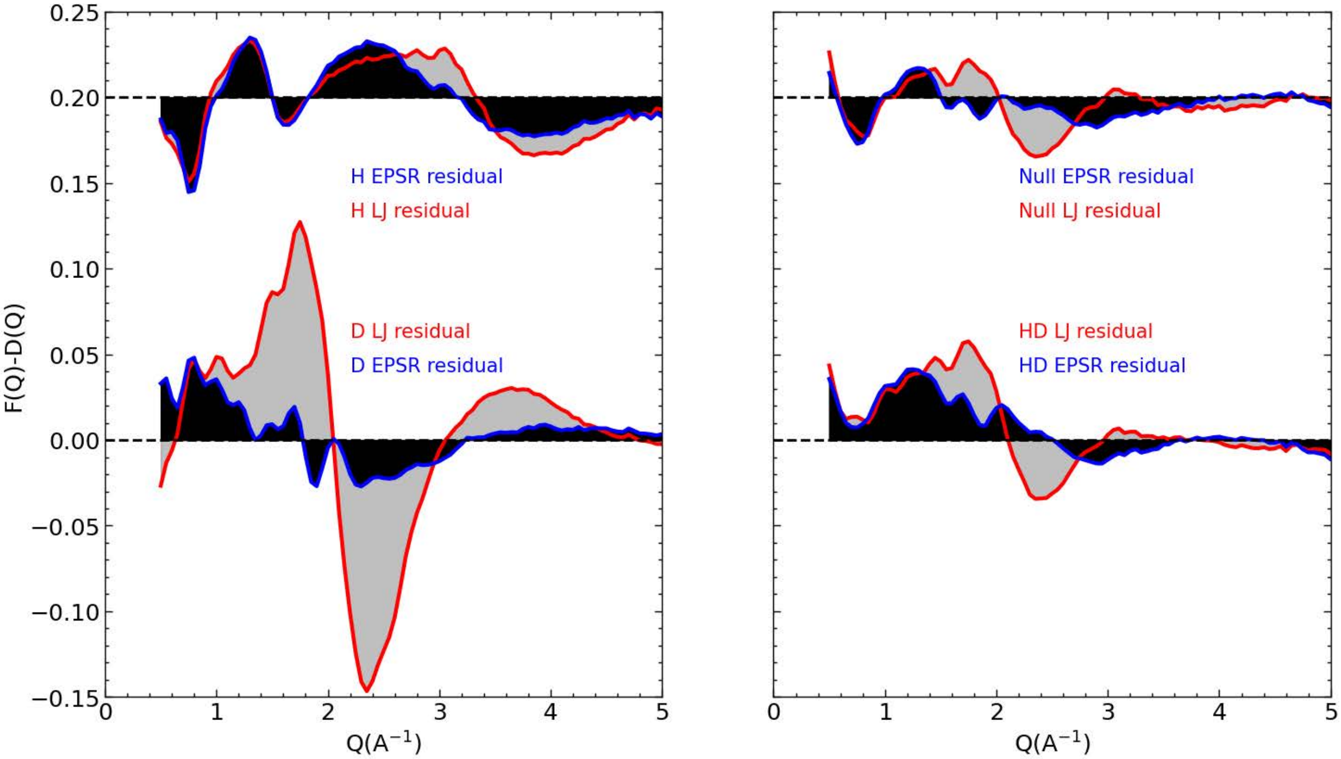

Fig. S28. A comparison of the residuals in the GPG in aqueous urea system described in Fig. S25 between $0.5 < Q \leq 5$ Å^{-1} so as to emphasise the difference.

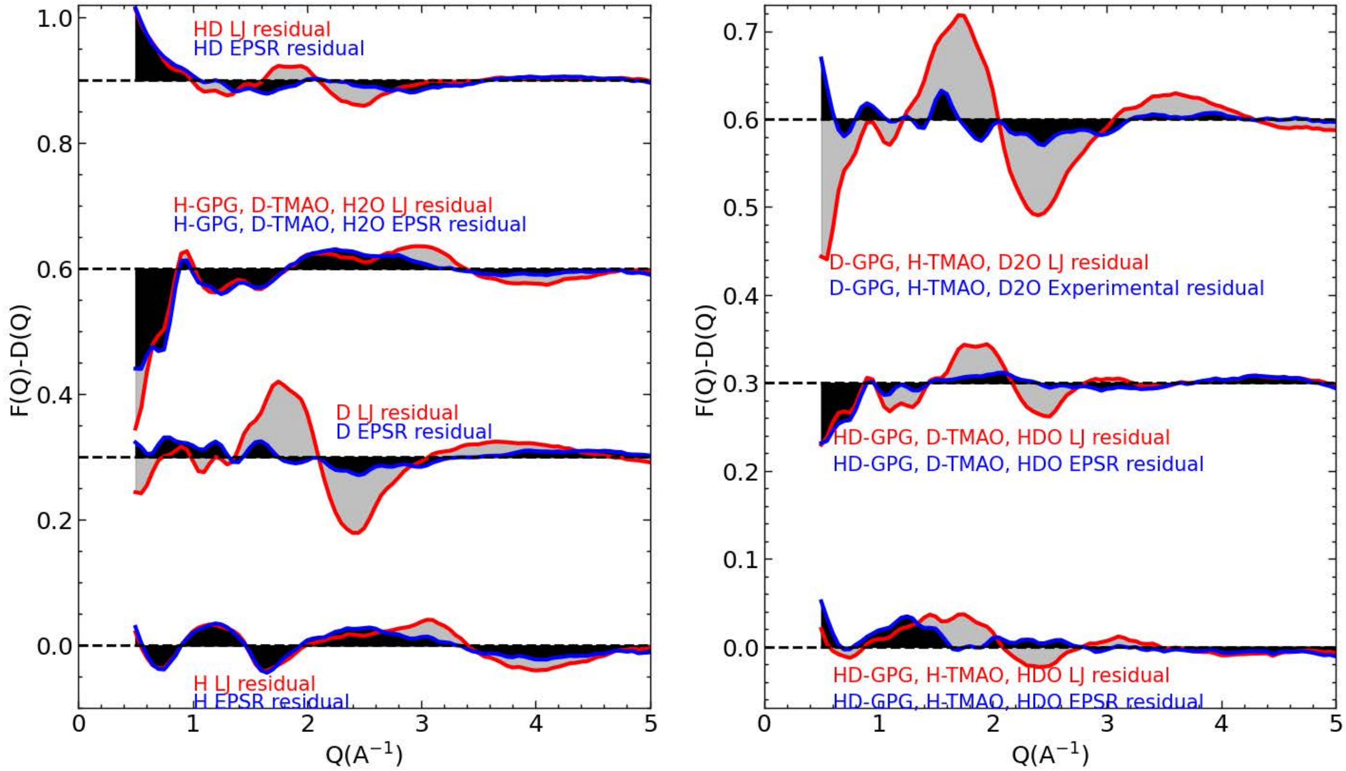

Fig. S29. A comparison of the residuals in the GPG in aqueous TMAO system described in Fig. S26 between $0.5 < Q \leq 5$ Å^{-1} so as to emphasise the difference.

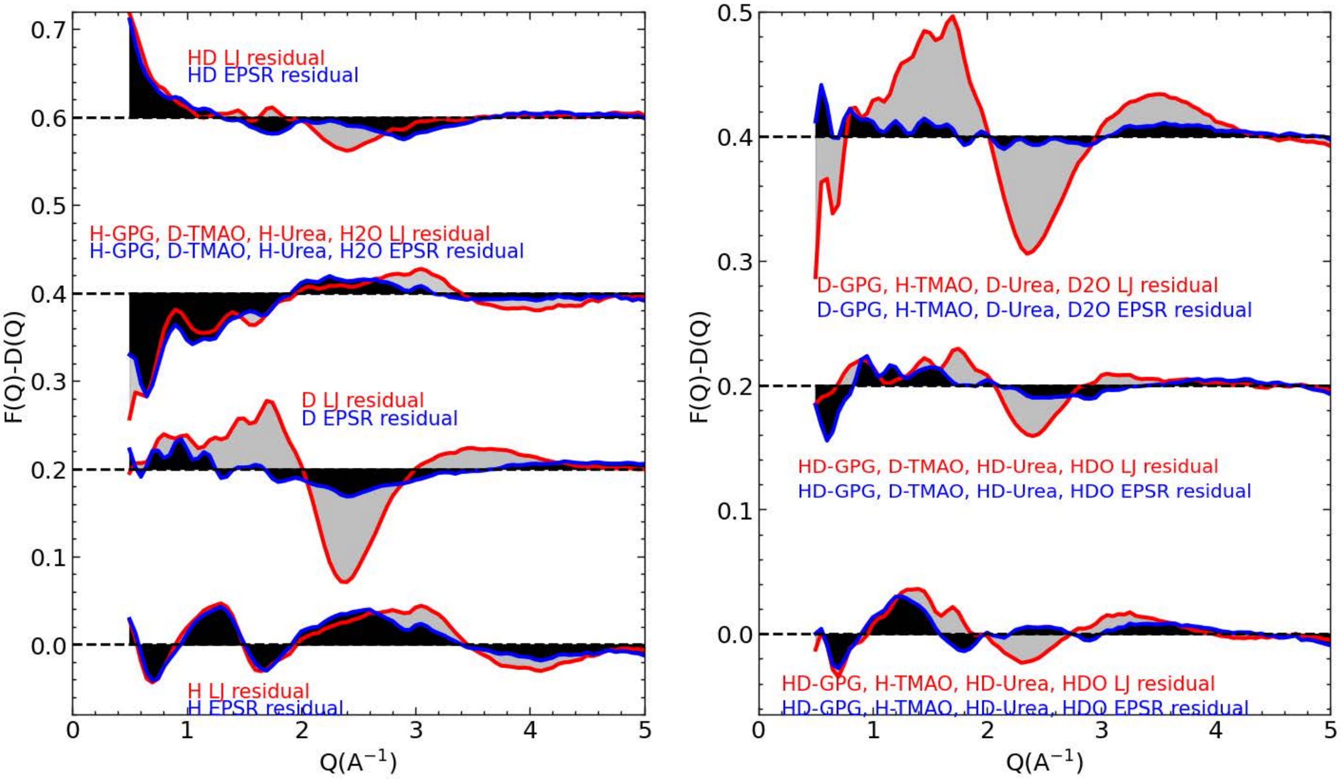

Fig. S30. A comparison of the residuals in the GPG in aqueous urea-TMAO system described in Fig. S27 between 0.5 < Q $\leq$ 5 Å^{-1} so as to emphasise the difference.

Table S1. Urea and TMAO preference ratios in binary and ternary solutions supporting Fig. 3 in the main text.

	**upr**		**Tpr**	
Peptide Atom	Urea	Urea + TMAO	TMAO	Urea + TMAO
N1	0.99	-0.20	0.20	0.10
N2	0.07	-0.17	-0.42	-0.52
N3	1.05	1.02	0.65	0.49
Nr	0.14	-0.08	0.11	-0.23
O1	0.61	-0.84	-0.66	-0.71
O2	-0.02	-0.66	-0.26	-0.52
O3	0.67	-0.49	-0.22	-0.23
C	0.11	-0.17	-0.33	-0.44
C1	0.17	-0.50	-0.35	-0.39
Cg	0.20	-0.03	-0.17	-0.34
Cr	-0.09	-0.43	-0.15	-0.49

Table S2. A comparison of the Urea Preference Indices of GPG's atoms in aqueous urea and aqueous urea-TMAO derived from series of Monte Carlo based simulations utilising a Lennard Jones potential (LJ) and an empirical potential (EPSR). The empirical potential is derived from the difference between the simulated D(Q) and the experimental F(Q). This data underlies the image in Fig. S10. The standard error was calculated using $\sqrt{2}$ given the small sample size. Although the sample size is small, and the molecular trajectory of some of the simulations is limited, certain trends are apparent which should give the reader some confidence in the reproducibility of the results. We note that the accumulation of urea, particularly around the amide group is consistent in the LJ, and EPSR models of the aqueous urea system, and is particularly pronounced in the EPSR model. The TMAO-induced urea depletion is consistently reproduced only with EPSR. It is evident across all models, that there is a high variance in the UPR around the carbonyl atoms. We suggest that this emphasises the sensitivity of the solvation structure to a peptide structure that is characterised by steric interactions. In the case of the carbonyl groups, their oxygen atoms are rotated into regions that are likely to be either readily accessible or otherwise to solute molecules. The extent then of a peptide-solute interaction is controlled in part by bond energy and by their mutual geometry, and the data in this table reflects that.

GPG-urea-water

	LJ only						With Empirical Potential					
**Atom-type**	**Run 1**	**Run 2**	**Run 3**	**Mean**	**SD**	**Std Error**	**Run 1**	**Run 2**	**Run 3**	**Mean**	**SD**	**Std Error**
Cumulative Iterations	933	2537	1662				2222	1982	32862			
N1	0.32	0.04	0.11	0.16	0.12	0.08	0.72	0.43	0.99	0.71	0.23	**0.16**
N2	0.01	-0.06	-0.25	-0.10	0.11	0.08	-0.02	-0.06	0.07	0.00	0.05	0.04
N3	0.38	0.25	0.00	0.21	0.16	0.11	0.32	0.45	1.05	0.61	0.32	**0.23**
Nr	-0.01	0.13	-0.05	0.02	0.08	0.05	-0.02	0.01	0.14	0.04	0.07	0.05
O1	0.34	0.31	-0.28	0.12	0.28	0.20	0.30	-0.23	0.61	0.23	0.35	**0.25**
O2	-0.10	-0.06	-0.12	-0.09	0.02	0.02	0.05	0.09	-0.02	0.04	0.05	0.03
O3	-0.05	-0.39	0.25	-0.06	0.26	0.18	0.23	-0.13	0.67	0.26	0.33	**0.23**
C	0.07	-0.04	-0.20	-0.06	0.11	0.08	0.09	-0.19	0.11	0.00	0.14	0.10
Cg	0.05	0.04	-0.11	0.00	0.07	0.05	0.12	0.01	0.17	0.10	0.07	0.05
C1	0.09	-0.02	-0.23	-0.06	0.13	0.09	0.09	-0.14	0.20	0.05	0.14	0.10
Cr	-0.16	-0.09	-0.27	-0.17	0.08	0.05	-0.20	-0.13	-0.09	-0.14	0.05	0.03

GPG-urea-TMAO-water

	LJ only						With Empirical Potential					
**Atom-type**	**Run 1**	**Run 2**	**Run 3**	**Mean**	**SD**	**Std Error**	**Run 1**	**Run 2**	**Run 3**	**Mean**	**SD**	**Std Error**
Cumulative Iterations	400	1500	1851				371	1739	24539			
N1	0.63	0.02	-0.28	0.12	0.38	0.27	-0.11	-0.32	-0.20	-0.21	0.09	0.06
N2	-0.26	-0.23	-0.10	-0.20	0.07	0.05	-0.35	-0.22	-0.17	-0.25	0.08	0.05
N3	0.25	0.12	0.33	0.23	0.09	0.06	0.29	-0.30	1.02	0.34	0.54	**0.38**
Nr	0.01	-0.01	0.02	0.01	0.01	0.01	-0.09	-0.05	-0.08	-0.07	0.02	0.01
O1	0.42	0.14	-0.33	0.08	0.31	0.22	0.25	-0.18	-0.84	-0.26	0.45	**0.32**
O2	-0.06	-0.15	0.24	0.01	0.17	0.12	-0.02	0.09	-0.66	-0.20	0.33	**0.23**
O3	-0.06	-0.04	0.14	0.01	0.09	0.06	-0.05	0.64	-0.49	0.03	0.47	**0.33**
C	-0.17	-0.16	-0.17	-0.17	0.00	0.00	-0.18	-0.10	-0.17	-0.15	0.04	0.03
C1	-0.01	-0.09	0.04	-0.02	0.05	0.04	-0.35	-0.32	-0.03	-0.23	0.14	0.10
Cr	-0.31	-0.09	-0.32	-0.24	0.11	0.08	-0.40	-0.17	-0.43	-0.33	0.12	0.08
Cg	-0.06	-0.10	-0.12	-0.09	0.02	0.02	-0.17	-0.04	-0.50	-0.24	0.19	0.14

Table S3. Molar ratios and isotopic components of the binary and ternary samples used in the neutron scattering experiment.

GPG-urea-water 01:06:58	GPG-TMAO-water 01:03:58	GPG-TMAO-urea-water 1:3:6:58
H-GPG, H-urea, H_2O	H-GPG, H-TMAO, H_2O	H-GPG, H-TMAO, H-urea, H_2O
D-GPG, D-urea, D_2O	D-GPG, D-TMAO, D_2O	D-GPG, D-TMAO, D-urea, D_2O
HD-GPG, HD-urea, HDO	HD-GPG, HD-TMAO, HDO	HD-GPG, HD-TMAO, HD-urea, HDO
HD-GPG, HD-urea, Null water	D-GPG, H-TMAO, D_2O	D-GPG, H-TMAO, D-urea, D_2O
	H-GPG, D-TMAO, H_2O	H-GPG, D-TMAO, H-urea, H_2O
	HD-GPG, H-TMAO, HDO	HD-GPG, H-TMAO, HD-urea, HDO
	HD-GPG, D-TMAO, HDO	HD-GPG, D-TMAO, HD-urea, HDO

Note: HD describes a number equality of protium and deuterium isotopolgues except HD GPG where HD references the 6 exchangeable hydrogen atoms i.e., each HD GPG molecule has 14 protium and 3 deuterium atoms. The null water sample contains by number 64% protium, and 36% deuterium isotopologues, a composition with no overall coherent neutron scattering effect.

Table S4. Assignment of Lennard Jones parameters to water's atom types in EPSR. The SPC/E model was adopted which features a bond angle of 109.47° and a bond length of 1.0 Å. This was changed to 104.2° and 0.976 Å in common with other diffraction studies (4, 5).

Molecule	Formula	Atom typw	No.	ϵ kJ/mol	σ Å	e e	Total e e
Water	H_2O	Ow	1	0.650	3.166	-0.8476	-0.8476
		Hw	2	0.000	0.000	0.4238	0.8476
							0.0000

Table S5. Assignment of Lennard Jones parameters to urea's atom types in EPSR(6).

Molecule	Formula	Atom type	No.	ϵ kJ/mol	σ Å	e e	Total e e
Urea	CH_4N_2O	Cu	1	0.439	3.75	0.142	0.142
		Hu	4	0.000	0.000	0.333	1.332
		Nu	2	0.711	3.250	-0.542	-1.084
		Ou	1	0.878	2.960	-0.390	-0.390
							0.000

Table S6. **Assignment of Lennard Jones parameters to TMAO's atom types in EPSR (7)**

Molecule	Formula	Atom type	No.	ϵ kJ/mol	σ Å	e e	Total e e
TMAO	C_3H_9NO	CT	3	0.390	3.70	-0.26	-0.78
		HT	9	0.065	1.80	0.11	0.99
		NT	1	0.711	3.250	0.44	0.44
		OT	1	0.585	3.080	-0.65	-0.65
							0.000

Table S7. **Assignment of Lennard Jones parameters to GPG's atom types in EPSR.(2, 3)**

Molecule	Formula	Atom type	No.	ϵ kJ/mol	σ Å	e e	Total e
GPG	$C_9H_{17}N_4O_3$.Cl	O1	1	0.50208	3.0291	-0.53	-0.53
		O2	1	0.50208	3.0291	-0.53	-0.53
		O3	1	0.50208	3.0291	-0.53	-0.53
		Nr	1	0.83680	3.2963	-0.27	-0.27
		N1	1	0.83680	3.2963	-0.49	-0.49
		N2	1	0.83680	3.2963	-0.62	-0.62
		N3	1	0.83680	3.2963	-0.30	-0.30
		C	3	0.37656	3.5636	0.53	1.59
		C1	1	0.15481	3.9645	0.05	0.05
		Cg	2	0.23012	3.8754	0.13	0.26
		Cr	3	0.23012	3.8754	-0.18	-0.54
		Hn1	1	0.19246	0.4000	0.31	0.31
		Hn2	1	0.19246	0.4000	0.30	0.30
		Hn3	3	0.19246	0.4000	0.33	0.99
		Hn4	1	0.19246	0.4000	0.32	0.32
		Hm	11	0.09205	2.3520	0.09	0.99
		Cl	1	0.62760	4.0447	-1.00	-1.00
							0.00

References

1. AK Soper, Partial structure factors from disordered materials diffraction data: An approach using empirical potential structure refinement. *Phys. Rev. B - Condens. Matter Mater. Phys.* **72**, 104204 (2005).
2. Nicola Steinke, R J. Gillams, L Carlos Pardo, C D. Lorenz, S E. McLain, Atomic scale insights into urea–peptide interactions in solution. *Phys. Chem. Chem. Phys.* **18**, 3862–3870 (2016).
3. M Di Gioacchino, F Bruni, MA Ricci, GPG-NH2 solutions: A model system for β-turns formation. Possible role of trehalose against drought. *J. Mol. Liq.* **335**, 116514 (2021).
4. F Meersman, D Bowron, AK Soper, MH Koch, An X-ray and neutron scattering study of the equilibrium between trimethylamine N-oxide and urea in aqueous solution. *Phys. Chem. Chem. Phys.* **13**, 13765–13771 (2011).
5. AK Soper, The radial distribution functions of water and ice from 220 to 673 K and at pressures up to 400 MPa. *Chem. Phys.* **258**, 121–137 (2000).
6. AK Soper, EW Castner, A Luzar, Impact of urea on water structure: A clue to its properties as a denaturant? *Biophys. Chem.* **105**, 649–666 (2003).
7. F Meersman, D Bowron, AK Soper, MH Koch, Counteraction of urea by trimethylamine N-oxide is due to direct interaction. *Biophys. J.* **97**, 2559–2566 (2009).
